# Dissolving the Dichotomies Between Online and Campus-Based Teaching: a Collective Response to *The Manifesto for Teaching Online* (Bayne et al. 2020)

**DOI:** 10.1007/s42438-021-00259-z

**Published:** 2021-10-29

**Authors:** Alison MacKenzie, Alexander Bacalja, Devisakti Annamali, Argyro Panaretou, Prajakta Girme, Maria Cutajar, Sandra Abegglen, Marshall Evens, Fabian Neuhaus, Kylie Wilson, Katerina Psarikidou, Marguerite Koole, Stefan Hrastinski, Sean Sturm, Chie Adachi, Karoline Schnaider, Aras Bozkurt, Chrysi Rapanta, Chryssa Themelis, Klaus Thestrup, Tom Gislev, Alex Örtegren, Eamon Costello, Gideon Dishon, Michael Hoechsmann, Jackeline Bucio, Guadalupe Vadillo, Melchor Sánchez-Mendiola, Greta Goetz, Helder Lima Gusso, Janine Aldous Arantes, Pallavi Kishore, Mikkel Lodahl, Juha Suoranta, Lina Markauskaite, Sara Mörtsell, Tanya O’Reilly, Jack Reed, Ibrar Bhatt, Cheryl Brown, Kathryn MacCallum, Cecile Ackermann, Carolyn Alexander, Ameena Leah Payne, Rebecca Bennett, Cathy Stone, Amy Collier, Sarah Lohnes Watulak, Petar Jandrić, Michael Peters, Lesley Gourlay

**Affiliations:** 1 School of Social Sciences, Education and Social Work, Queen’s University Belfast, Belfast, BT7 1HL UK; 2 Melbourne Graduate School of Education, University of Melbourne, Melbourne, Australia; 3 Department of Accounting and Finance, Lancaster University, Lancaster, UK; 4 National Higher Education Research Institute (IPPTN), Universiti Sains Malaysia, Penang, Malaysia; 5 Dublin City University, Dublin, Ireland; 6 Department of Arts, Open Communities & Adult Education, University of Malta, Msida, Malta; 7 Division of Digital Learning, KTH Royal Institute of Technology, Stockholm, Sweden; 8 School of Architecture, Planning and Landscape, University of Calgary, Calgary, Canada; 9 Science Policy Research Unit, University of Sussex, Brighton, UK; 10 Educational Technology & Design, Department of Curriculum Studies, University of Saskatchewan, Saskatoon, Canada; 11 Faculty of Education and Social Work, University of Auckland, Auckland, New Zealand; 12 Digital Learning, Deakin University, Melbourne, Australia; 13 Department of Education, Umeå University, Umeå, Sweden; 14 Open Education Faculty, Distance Education Department, Anadolu University, Eskişehir, Turkey; 15 Faculty of Social Sciences and Humanities, Universidade Nova de Lisboa, Lisbon, Portugal; 16 Department of Education and Lifelong Learning, Norwegian University of Science and Technology, Trondheim, Norway; 17 Danish School of Education, Aarhus University, Aarhus, Denmark; 18 Centre for Educational Development, Aarhus University, Aarhus, Denmark; 19 Department of Applied Educational Science, Umeå University, Umeå, Sweden; 20 Dept of Education, Ben Gurion University, Beersheba, Israel; 21 Lakehead University Orillia, Heritage Place, 1 Colborne Street West Orillia, Orillia, ON L3V 7X5 Canada; 22 Online High School & MOOC, Open University, Educational Innovation and Distance Education Office, Universidad Nacional Autónoma de México, Mexico City, Mexico; 23 Open University, Educational Innovation and Distance Education Office, Coordinator, Universidad Nacional Autónoma de México, Mexico City, Mexico; 24 English Department, Faculty of Philology, University of Belgrade, Studentski trg 3, 11000 Belgrade, Serbia; 25 Department of Psychology, Federal University of Santa Catarina, Florianopolis, Brazil; 26 Institute of Sustainable Industries and Livable Cities (ISILC), College of Arts and Education, Victoria University, Footscray Park Campus, Footscray, Australia; 27 Jindal Global Law School, O. P. Jindal Global University, Sonipat, India; 28 Institute for Danish Game Development, Dania Academy, Grenaa, Denmark; 29 Faculty of Social Sciences, Tampere University, Tampere, Finland; 30 The University of Sydney, Sydney, NSW 2006 Australia; 31 Department of Education, University of Gävle and Umeå University, Umeå, Sweden; 32 Department of Education, Stockholm University, Stockholm, Sweden; 33 Moray House School of Education and Sport, The University of Edinburgh, Edinburgh, Scotland; 34 School of Educational Studies and Leadership, University of Canterbury, Private Bag 4800, Christchurch, New Zealand; 35 Future Learning and Development, University of Canterbury, Private Bag 4800, Christchurch, New Zealand; 36 FarNet, 230 Port Marsden Highway, Ruakākā, RD1, Whangarei, 0171 New Zealand; 37 School of Arts and Education, Deakin University, Melbourne, Australia; 38 Kulbardi Aboriginal Centre, Murdoch University, Perth, Australia; 39 University of Newcastle, Newcastle, Australia; 40 National Centre for Student Equity in Higher Education (NCSEHE), Curtin University, Perth, Australia; 41 Office of Digital Learning and Inquiry, Office of the Provost, Middlebury College, Middlebury, VT USA; 42 Office of Digital Learning and Inquiry, Middlebury College, Middlebury, VT USA; 43 Zagreb University of Applied Sciences, Zagreb, Croatia; 44 University of Wolverhampton, Wolverhampton, UK; 45 Beijing Normal University, Beijing, China; 46 University College London Institute of Education, London, UK

**Keywords:** Collective response, *Manifesto for teaching online*, Digital learning, Campus learning, Distant learning, Covid-19, Postdigital

## Abstract

This article is a collective response to the 2020 iteration of *The Manifesto for Teaching Online*. Originally published in 2011 as 20 simple but provocative statements, the aim was, and continues to be, to critically challenge the normalization of education as techno-corporate enterprise and the failure to properly account for digital methods in teaching in Higher Education. The 2020 *Manifesto* continues in the same critically provocative fashion, and, as the response collected here demonstrates, its publication could not be timelier. Though the *Manifesto* was written before the Covid-19 pandemic, many of the responses gathered here inevitably reflect on the experiences of moving to digital, distant, online teaching under unprecedented conditions. As these contributions reveal, the challenges were many and varied, ranging from the positive, breakthrough opportunities that digital learning offered to many students, including the disabled, to the problematic, such as poor digital networks and access, and simple digital poverty. Regardless of the nature of each response, taken together, what they show is that *The Manifesto for Teaching Online* offers welcome insights into and practical advice on how to teach online, and creatively confront the supremacy of face-to-face teaching.

## Introduction (Alison MacKenzie)

In February 2021, Petar Jandrić approached me to ask if I would coordinate a call for responses to *The Manifesto for Teaching Online* (Bayne et al. 2020). This book is the third iteration of the series that began with the online ‘Manifesto for Teaching Online’ in 2011, with a follow-up in 2016^1^. The aims of the manifestos were, and continue to be, to critically challenge the normalization of education as techno-corporate enterprise and the failure to properly account for digital methods in teaching in Higher Education (HE). This was an opportunity I couldn’t turn down. The call went out in late February, and by June, it yielded responses presented in this paper. Clearly, *The Manifesto* offers a much-needed perspective that calls for debate — and in the time of the Covid-19 pandemic, that debate could not be timelier.

It needs hardly be said that 2020/2021 was the period during which online teaching became a necessity. As the director of a master’s programme on Special Needs Education and Inclusion, and who has a keen interest in social justice, how to teach online in ways that were creative and inclusive was of huge concern to me at the start of the pandemic. I was also concerned about supporting students who became anxious, fearful, and insecure, many of whom lost their jobs (temporary teaching contracts), had caring responsibilities, who found themselves unable, suddenly to conduct research, or who had to compete for digital access. They were also very worried about the transition from campus-based learning to online learning which, many of my students believed, would be inferior — and in too many cases, this proved to be the case.

In the process of moving (or scrambling) online, digital learning intensified, diversified, and surprised. Online teaching surprised because educators found that they could, after all, teach remotely and that it could be as effective as face-to-face teaching, if used well and creatively. Universities were suddenly confronted with the necessity of providing structures that would allow their students to study remotely and very quickly found the capacity and flexibility to do this, a capacity they had had all along but for reasons of competition, effort, interest, or lack of commitment to equity and justice (for marginalized groups, that is) chose not to provide.

The move to online teaching greatly benefited people with some types of disability, such as immunocompromised students or students with mobility or respiratory problems. For others, such as the visually or hearing impaired, blind, and deaf, and autistic students, it represented (and may continue to represent) significant challenges. These challenges include technology that has not advanced to a stage where it can be used be effectively, such as online stenotype services/captions for the deaf or hearing impaired. A large number of students are also affected by digital poverty. Research by UK’s National Union of Students (2020) found that 27% of university students could not access online learning during the pandemic, and students with disabilities and low socio-economic backgrounds were most affected.

This very brief analysis provides the background to concerns about marginalization and exclusion of minority groups on campus and online. Questions about the (post)digital have been richly explored in Postdigital Science and Education journal and book series^2^, and recent volumes have explored how Covid-19 has impacted on teaching and learning^3^, including collective testimonials articles ‘Teaching in the Age of Covid-19’ (Jandrić et al. 2020), ‘Teaching in the Age of Covid-19: 1 Year Later’ (Jandrić et al. 2021a), and the follow-up analysis article (Jandrić et al. 2021b). Like these contributors, I fretted over how to teach in this new environment under pandemic conditions, and many of the responses gathered here inevitably reflect similar concerns.

Written before the Covid-19 pandemic, *The Manifesto for Teaching Online* (Bayne et al. 2020) raises many questions that extend beyond our pandemic-related concerns, including those concerned with philosophy and the practices of critical posthumanism. *The Manifesto* developed from a short web provocation, a decade, almost, of conversations in and around Edinburgh University’s Centre for Research in Digital Education^4^, to a fully developed book. *The Manifesto for Teaching Online* (Bayne et al. 2020) represents the essence of these conversations, and critical conversations, being what they are, should be continued, and so this article is Postdigital Science and Education community’s contribution to extending the work started by Bayne and colleagues well into the future.

## Sociomaterial Invitations and Reassurances: The Rich Possibilities of Online Teaching

### Sociomaterial Entanglements and Dialogic Imperatives (Alexander Bacalja)

Online teaching became the modus operandi for educators across the globe in 2020 as the Covid-19 pandemic forced formal institutional teaching and learning into the digital realm. Those faced with this sudden and violent change were forced into experiences often characterized by sociomaterial entanglement, where the pursuit of dialogic forms of teaching required mediation through digital technologies.

Those advocating for dialogic approaches to education argue that dialogic teaching harnesses the power of talk to stimulate and extend a learner’s thinking and advance their learning and understanding (Alexander 2020). It’s not just any talk that is valued, but rather talk that mediates the cognitive and cultural spaces between individuals (Mercer et al. 1999; Nystrand et al. 2001). However, the burgeoning development of digital technology for educational purposes has raised concerns about new ways to access, use, and spread information (Jandrić 2017; Selwyn et al. 2020), with preliminary research suggesting that the impact on classroom dialogue has not been benign (Major et al. 2018).

*The Manifesto for Teaching Online* (Bayne et al. 2020) suggests that a sensitivity to the dynamic entanglements of digital education, especially at the nexus of social and material factors, is vital for seeing past approaches that ignore how the sociomaterial dialectic can offer pathways forward. One example of educators seeking to make sense of the role of the dialogic in their sudden shift to online teaching serves to highlight the complexity of these entanglements.

In mid-2020, I was working with a large high school in suburban Melbourne to support their leadership team to adopt literacy practices that would recognize oracy as central to teaching and learning. Government-imposed lockdowns forced all teaching into the online sphere. The shift produced pedagogies inconsistent with our intended focus on oracy and the dialogic. Trust in the professionalism of staff was undermined by directives from leadership that students are not allowed to meet in online meeting rooms without a teacher present. Concerns about intrusion into students’ private lives, through webcams projecting directly into bedrooms, led to an acceptance, if not encouragement, of students switching off their cameras. The consequence was a sea of black screens and a reluctance on the part of students to participate in social learning. This in turn led teachers to avoid activities which encouraged sociality. The material circumstances of the technology, coupled with concerns about the social, blurred public/private boundaries, created entanglements that ultimately promoted didactic forms of online teaching.

As Bayne et al. (2020) have argued, teaching online is always situationally contingent, and inherently multiple, producing realities that have often underestimated the implications for access and equity. In his work on the conditions that physical spaces place on individuals, Bourdieu (1999: 128) states that ‘[a]t the risk of feeling themselves out of place, individuals who move into a new space must fulfil the conditions that that space tacitly requires of its occupants’. If the digitally mediated environments that we construct for students privilege didactic forms of knowledge (re)production, then we should not be surprised when learners respond by turning off their screens and refusing to engage in talk. On the other hand, if these new spaces are redesigned as dialogic spaces, where talk is privileged as a tool for responsive interaction, then the technologies of teaching online might become the tools for high-quality learning that we all need them to be.

### Digital Learning. Don’t Bother Teaching Students. Give Them Tools to Lead to New Ways of Thinking (Devisakti Annamali)

Digital learning? Why now? These questions linger in many educators’ minds. As the world evolves towards disruptive technology, graduate students entering the labour market are expected to be highly equipped with digital skills and knowledge. Our argument is that educators can’t fully rely on traditional teaching and learning methods anymore. In addition, the Covid-19 pandemic saw the teaching and learning process in higher education shift online.

Teaching based on technology has attracted much attention for the past ten years to enhance learner’s learning and engagement, while higher education around the globe has evolved its teaching and learning techniques. Today’s learners have grown up to be more tech-savvy than before, and as our learners evolve as digital citizens, so must our educators. A good educator in higher education must be able to integrate digital technology to fulfil learners' needs in higher education (Amhag et al. 2019).

Today’s learners are digitally literate but not yet digitally fluent. A learner who is digitally literate understands and uses the basic functions of the digital well, while a learner who is digitally fluent is technically proficient and has intellectual and social competencies (MacKenzie 2016). There are a wide range of digital tools that can be used by educators in the classroom. Tablets, short films, power point, and chalkboards are some examples of digital tools which could attract learners towards teaching and at the same time learn new technology skills.

Technology tends to change the way we think. In order to survive the modern world, learners need life-long learning skills, to widen knowledge, adapt to changes, and successfully manage and produce information (Gökçearslan et al. 2019). Efficient use of technology in a learning environment improves learners’ study skills and ability to implement real-life situations and gain critical and problem-solving thinking skills. It positively affects learners’ professional life after graduation (Bimrose et al. 2014). Digital learning skills allow learners to be creative, to analyse, and evaluate information. Learners’ involvement in discussions via technology platforms can support their higher-level thinking processes and provide more opportunities for them to participate, cooperate, and interact.

*The Manifesto for Teaching Online* (Bayne et al. 2020) does not argue that educators should ignore traditional teaching and learning, but that they integrate digital tools into their teaching and learning methods to pave the way for new ways of thinking among students.

### Online Can Be the Privileged Mode for Large Classes (Argyro Panaretou)

During the last decades, a number of socio-politico-economic reasons have resulted in an increase in the demand for university degrees offered by management and business schools. Without a proportional increase in government subsidies, the only way for the universities to cope with the new student numbers was to increase the physical space to accommodate larger classes. Therefore, the first ‘distance’ learners in management/business schools appeared a while ago, sitting at the back of a huge lecture theatre, with little interaction.

At the start of Covid-19 pandemic, ‘traditional’ face-to-face teaching had to be substituted, at very short notice, with online teaching. My first thought was that we were going to offer our students a low-quality alternative, but I very soon realized that online education is neither isolating nor demotivating.

I was asked to pre-record lectures for my large MSc class of 100 + international students. With the use of a digital whiteboard, the whole experience reminded me of a lecture delivered in the class, with some extra advantages. All my students could hear me well, could see what I was writing on the whiteboard clearly, and most importantly, they could cover the material at their own pace. Subtitles were included in each video, reducing the language barrier, and plenty of questions were uploaded in the course website for the students to check their understanding on the delivered material. Weekly synchronous sessions in small groups were all about discussing the material and answering students’ questions. This gave us more time for dialogue than face-to-face delivery, and the technology provided new affordances for this, with questions and ideas also posted in the chat and the course website. A short online test every two weeks kept the students engaged with the material and provided them with instant feedback on their progress.

Overall, online delivery was a very positive experience for my students and made me, a traditional educator, realize that technology can help us to achieve equitable quality and more inclusive education. With fewer constraints on physical space, universities can expand the number of scholarships available to developing countries. We can use the advantages technology offers (including remote learning) to ensure that people with disabilities and caring responsibilities have equal access to university and address gender disparities in education. Finally, but importantly, online teaching decreases the need for students to travel frequently to access university education, contributing to combating climate change.

### A Perspective from the Periphery: *The Manifesto* as a Welcome Sign (Prajakta Girme)

I am from India and the politically charged atmosphere remains in the country, with alleged use of surveillance to root out dissent among the people (Perrigo 2021). *The Manifesto for Teaching Online* (Bayne et al. 2020) made me question where normalization of surveillance starts. Is it in the classrooms? Is it earlier with exposure to social media outside of it? Do universities add to this budding mindset of surveillance being the norm? Is it to the extent that people start accepting surveillance on a wider, social scale? The book has left me with questions but with those questions, it has given me words for expression of injustice and immorality. The questions that started through digital visibility in pedagogy have evolved to resonate at a very personal albeit political level.

*The Manifesto* centres its critical argument against surveillance in higher education on Lyon (2017: 835) observation that ‘surveillance ought not merely be of people … so much as for people – and thus be practiced carefully and held to account’. A surveillance society is one where surveillance is understood as being done to people by agencies. Surveillance culture, by contrast, is ‘widespread compliance with surveillance’ (Lyon 2017: 828). The book promotes dialogue, giving a common language with which to dissect, examine, and attempt to encapsulate the role of society heading towards a potentially problematic surveillance culture. In its argument of pedagogy, it highlights the greater ethical dilemmas of digital visibility where distrust is sown early in the student’s lifestyle perhaps making them more compliant to it outside of the university setting. Before we question the existence of surveillance culture, there are questions we should ask ourselves as part of a possible surveillance society; of what we might allow an institution to normalize and of what we might unwittingly enable enforcement.

As someone new to the field of education research, I felt at times crowded by convoluted, jargon-filled, cleverer-than-thou academic literature. *The Manifesto for Teaching Online* (Bayne et al. 2020) stood out for me as a lucid, accessible read. It practices what it preaches in its easy to comprehend, intelligible style. Personally, I have always thought that the veins of education run stale with confining, traditional approaches to teaching and learning.

**Fig. 1:**
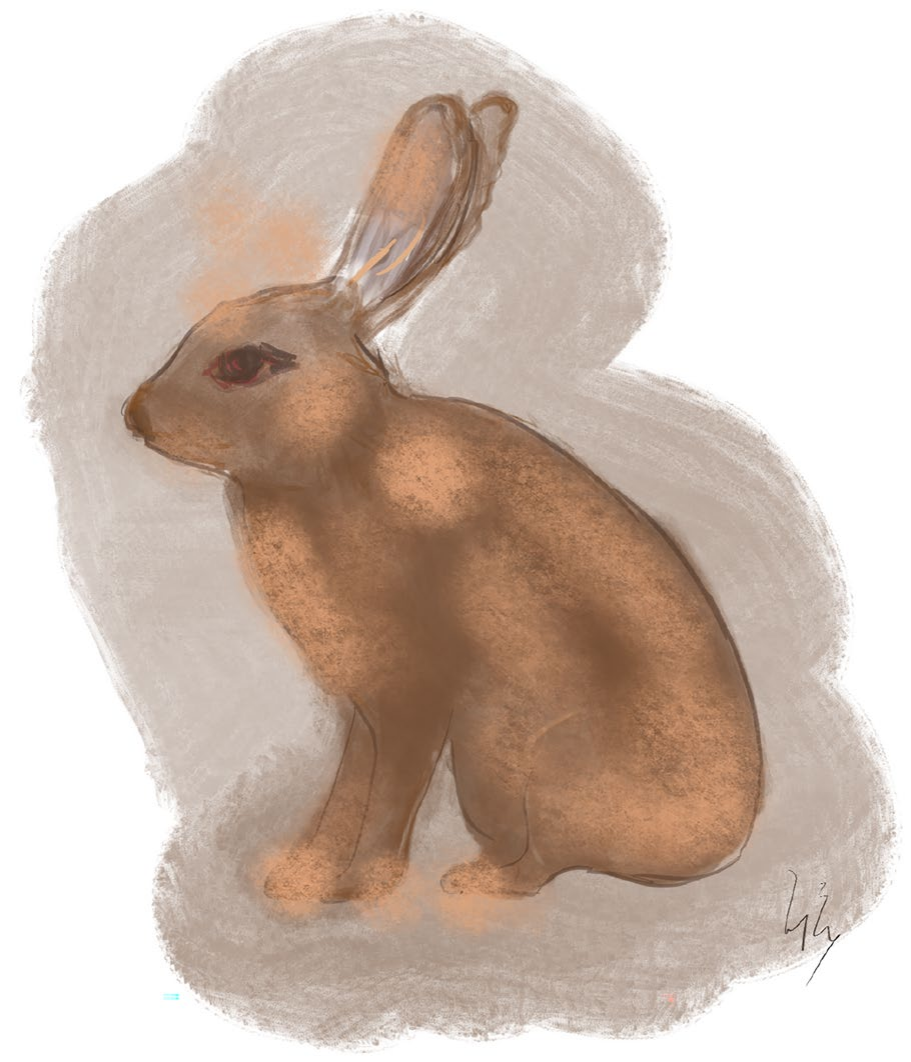
*The Manifesto* and multimodality

As a child, I loved illustrated encyclopaedias. In that sense, *The Manifesto* resonated with me on multimodality. I contributed illustrations to academic articles (Costello et al. 2020; MacKenzie et al. 2021). *The Manifesto* encouraged me to push for a multimodal methodology for my PhD proposal, and I understood that varied forms of representation of academic knowledge were legitimate (Fig. 1). The book makes an argument for multimodality that rests on the disconnect between the traditionally text-centric channel of communication in research, and the world being studied, which ‘is visual, aural, tactile, multimodal, multidimensional, and polysemic’ (Andrews et al. 2012: 24). *The Manifesto for Teaching Online* critically probes at the burdens carried by the written word: ‘Text has been troubled’ (Bayne et al. 2020: xi).

### Inviting Criticality and Marginality in Teaching Online: the New Normal Education (Maria Cutajar)

*The Manifesto of Teaching Online* (Bayne et al. 2020) is a thought-provoking academic critique inviting deepened discernment and soul searching for teaching online. It calls to attention problematic themes and subthemes related to teaching online and related obscured beliefs, popular myths, binary thinking, and neoliberal narratives of the twenty-first century aspirations incorporating a lot of ‘learnification’ (Biesta 2009) but leaving teaching and learning undisturbed. Published just in time of the Covid-19 public health crisis which saw much teaching shifted online or nothing at all, this *Manifesto* invites criticality. The authors exemplify this by tracing back to the roots of issues and perspectives critically considering their trajectories into the present, bringing to the fore assumptions, presumptions, and mythical sensationalisms.

As the world looks forward beyond the global pandemic crisis whereby blended and online teaching are widely forecasted as the new normal, a critical mindset is important. The need of such a critical attitude heightens with recognition that terms like ‘networked learning’ and ‘transformative education’ are increasingly fashionable buzzwords; and fundamental humanistic principles of equity, social justice, inclusivity, and diversity are fast becoming empty but reigning jargon in teaching (and learning) online. An ongoing reflective and critical attitude appears to be a constructive option to hold on to as educators navigating the evolving digital education landscape. The call is to morph into critical being beyond critical thinking; that is, to go beyond critically thinking about the world to critical self-reflection/reflexivity and critical action in being part of the world (Barnett 1997; Dunne 2015). Rather than being merely an object of education, criticality serves as a guiding compass for education (and beyond). Arguably, criticality should be the new normal education.

Living up to and fostering criticality — to stop and think and possibly do things differently — demands strength and willingness to live perpetually on the periphery of what is mainstream. The margin provides space and possibility of a clear viewpoint of the changing landscape and the capacity to question the unquestionable and to articulate our sense of the world unimpeded (hooks 1989). It is from the margin that imprints, emergent topologies, trails, and trajectories are most likely to be discerned. The margin holds the keys that unlock the shackles of capitalist forces, political agendas, and competing and conflicting ideologies which increasingly seek to impress and oppress teaching online and digital education generally. Without discernment of what is at stake, and furthermore the embedding and surrounding contextual brokers playing for the stakes, there is no possibility to question that which in the mainstream appears unquestionable and irresistible. It is at the margin that resistance is possible (hooks 1989).

This is what *The Manifesto of Teaching Online* (Bayne et al. 2020) essentially calls for to remain relevant in the future. The landscape of digital education and teaching online is fast changing and warping, and just as rapid is the race to appropriation by big businesses (Williamson 2020). Caught on the frontline of this battlefield of appropriating forces on the ground are the teachers and learners. *The Manifesto* stands with these stakeholders at the heart of the educational enterprise, stakeholders who embrace the margin. Embracing the margin opens the stage for criticality as in critical being. It potentially opens cracks for light to flow through to see more clearly what it means and what it takes to positively advance practices (and theory) in the emergent educational landscape, which, in post-Covid-19 pandemic times, appears to be precipitating towards hybridity. It is this marginality and criticality that makes possible ambitions for a sustainable new normal education where equity, social justice, inclusion, and diversity are not reigning jargon, but reigning trends.

### Voices from the Digital Classroom (Sandra Abegglen, Marshall Evens, Fabian Neuhaus, Kylie Wilson)

The Covid-19 pandemic has forced the world to engage with the digital classroom. While online learning and teaching have been explored and practiced since the invention of computers and the World Wide Web, fully web-based universities only gained traction in the late 1990s. Online education has many benefits, such as opening new avenues for creative and innovative methods of delivering information. However, it also poses many challenges, for example, the dismantling of historical practices and shared tendencies surrounding in-person higher education. The closure of the world due to Covid-19 quickly and unexpectedly opened new discourses surrounding access to education and information. Data protection and Artificial Intelligence are areas that are debated, together with questions of what constitutes ‘inclusive practice’ and whether ‘diversifying’ ideas and software help.

*The Manifesto of Teaching Online* (Bayne et al. 2020) intends to serve as a resource and an inspiration for those teaching in online environments through the offering of twenty-one statements, crafted as an invitation for dialogue and interpretation. Although the bounds for discussion are endless, we have found one thing becoming more and more clear throughout the shift to emergency remote learning (Hodges et al. 2020): with any major change, we must also shift our ontology and epistemology, and the time is now to take a critical look at higher education.

*The Manifesto* reminds us that education is often seen and taught in a linear or singular frame of mind. Online education pressures us to push beyond this traditional mindset by its own nature of being digital. Education, whether in person or online, ought to be dynamic in its frameworks, structures, and delivery, as the world, too, is dynamic. Our world is ever changing; complexities must be embraced.

Within these complexities, there is the realization of our historical tendency to see face-to-face as the superior method of education. We now have a recognition of negative discourses that surround online education, yet when the pandemic began, we no longer had a choice. Is this simply because we have never had to do this before? Are we all simply intimidated by the infinite vastness and possibilities of online education? Are we not yet ready to take on the task of figuring out how to do this? *The Manifesto* proposes that the digital realm must be embraced and that there are many ways to get it right. In it, we find guidance as we now navigate through the wide range of methods of communication and learning deliveries that are made possible online.

As has been made apparent, interface shapes everything; learning has the potential to be defined by visual and interactive experience. *The Manifesto* argues that these should not be reduced to second-best, rather they should be diligently explored. Good education practices need to transcend all learning environments, whether in person or online, so shifting our mindset is proposed as a key factor and that engaging with what is possible and desirable in online education can inspire. As there is no textbook, and nothing is black and white, it is time to explore the grey, in-between spaces of possibility and create opportunity for meaningful change.

### Good Digital Education is Possible, But there are Challenges to Overcome (Katerina Psarikidou)

Good Digital Education is possible. This is the key message of *The Manifesto* (Bayne et al. 2020) that I would like to take forward in my attempt to contribute to a better future for online teaching. However, it can be challenging, especially when having to overcome narrow instrumentalist approaches to higher education, in which ‘the digital’ can turn into yet another mechanism for pursuing the post-industrial imaginary of knowledge-driven economic growth (Bayne et al. 2020; Psarikidou 2021). This is another significant point raised in *The Manifesto* with which I would like to critically engage in an attempt of identifying meaningful ways of overcoming it.

I am a lecturer for a fully online distance learning MSc programme, developed in collaboration with a specialist EdTech service provider. Teaching for this programme helped me challenge some of my pre-established othering assumptions of online teaching and appreciate the diverse and positive learning experiences that are opened for both teachers and learners (Beethan and Sharpe 2013). However, it has also made me anxious, particularly about the role of technologies in shaping my teaching practices, and of corporate partners in shaping processes of ‘transmission’ of academic knowledge to students (Ivencheva et al. 2020).

Reading *The Manifesto* has reassured me in a lot of different ways with regard to my experience of online teaching. Indeed, in many cases, online teaching can be reduced to ‘facilitation’, therefore carrying broader implications on the value and the autonomy of the academic as subject-matter expert (Bayne et al 2020). This can also be due to a prevailing techno-solutionist, infrastructure-first approach to digital learning (Bayne 2015), particularly present when EdTech becomes intermediaries of teacher-learner knowledge-transfer interactions. As suggested in *The Manifesto*, a more-than-human approach to teaching is prevalent (Pedersen 2015). It is, therefore, important for us teachers to critically reclaim our central role in such complex sociomaterial entanglements between people and technologies, through which universities are currently enacted (Urry 2007).

Universities are not just about physical spaces and infrastructures (and their fetishization); they are about people (and relations). And, indeed, this principle very much resonates with my online teaching practices as well as my strategies for overcoming some of the above challenges. It has helped me not only configure new relations of proximity, intimacy, and co-presence between me and my online students (Boden and Molotch 1994), but also value ‘distance’ and ‘difference’ as important drivers for enacting more inclusive forms of teaching based on more socio-culturally diverse communities of learning (Louie 2005).

Education is now so entangled with the digital these days. So, it is possibly about time to move beyond ‘campus’ vs. ‘distance’ or ‘online’ vs. ‘face-to-face’ binaries. This is particularly important if we want to overcome processes of commodification and instrumentalization of education which are common to both ‘campus’ and ‘distance’ contexts and can be key for configuring better educational futures both online and offline. *The Manifesto* provides the space for us fellow teachers to join voices and forces for prefiguring as well as enacting such futures.

## Calling Attention to Learnification and ‘Best’ Practice

### The Paradox of Learnification (Marguerite Koole)

It is understandable that educators would wish to perceive their work as learner centred. On the surface, it is a noble cause. At a deeper level, naïve adherence to the taken-for-granted language of learner-centredness perpetuates hegemonic practices that ultimately imperil the overall well-being of not only society, but also the materialities of which people and society are inherently a part. In economic terms, learnification offers a neo-liberal-friendly conception of learning as a task, achievement, or product. Teaching in this view is facilitation, an undervalued mechanism for students to consume knowledge or skills to participate in the labour market.

In practice and in rhetoric, learner-centredness sees the individual learner, the human subject, as the only entity that matters, a viewpoint that may be traced back to humanist philosophical roots. Barad (2007) challenges this type of human exceptionalism. For her, the primary ontological unit is not a thing or a person, but a phenomenon. In Barad’s ‘agential-realist account, discursive practices are specific material (re)configurings of the world through which the determination of boundaries, properties, and meanings is differentially enacted’ (148). Through intra-action, the social and material perform phenomena into being; specific intra-actions enact distinct ways of being. ‘It is through specific intra-actions that phenomena come to matter—in both senses of the word.’ (140) Rather than increasing transparency, a hyper-focus upon the learner obscures recognition of inherently entangled, co-constructing entities—such as teachers, communities, and other sociomaterial-digital elements.

Learnification appears entangled in paradoxes that require further examination. For example, while superficially promoting the learner to the highest status, learner-centredness instead leads to mistrust of both teachers and learners. As Bayne et al. (2020) remark, learnification de-professionalizes the role of the teacher while concomitantly implying that the learner is self-motivated and possesses fully developed cognitive and metacognitive skills to direct their own learning.

Yet, the learner cannot be trusted: in many institutions of higher education, learning analytics, plagiarism detection, and examination proctoring software are routinely implemented. Such regulatory mechanisms undermine the very idea of learner agency. As Bayne et al. (2020) suggest, the adoption of control and surveillance technologies erode trust, jeopardizing relationships between the learner, the teacher, and the institution. How do well-meaning teachers rationalize their desire to be learner-centred and their mistrust of learners? And how can educators move forward? As *The Manifesto* (Bayne et al. 2020: 186) indicates, there may be better ways to solve perceived problems such as focusing on learning and assessment design—particularly by ‘designing-out plagiarism’. Furthermore, Mulcahy (2012: 21) suggests that considering ‘pedagogy as an assemblage affords a sense of collective responsibility’.

Sociomaterial approaches can help educators de-centre the human and move towards a more relational, democratic, and inter-subjective collective activity (Parchoma 2016) in which other, no less important, entities, can come to matter and contribute to each other’s well-being—rather than becoming merely cogs in an economic wheel.

### Getting it ‘Right’ Online: Teaching Online as a Complex and Informed Practice (Stefan Hrastinski)

*The Manifesto for Teaching Online* (Bayne et al. 2020) might become even more important in post-pandemic higher education. While some describe the use of digital technologies during the pandemic as a great success, others point out that much of the teaching practices we have seen could be described as emergency remote teaching that is not informed by what is known about teaching online (Hodges et al. 2020). *The Manifesto* could serve as a source of inspiration in discussions on how we would like online teaching to develop during coming post-pandemic years.

In particular, I would like to comment on the first point of *The Manifesto*: ‘There are many ways to get it right online. “Best practice” neglects context.’ The authors challenge the notion of teaching being necessarily focused on pre-existing ‘objects’ of study and students as stable ‘learning’ subjects. Convincingly, it is argued that ‘the very idea of a single, immutable, “best practice” becomes untenable: online and off, there are many ways to get it right’ (Bayne et al. 2020: 17). While I certainly agree that there are many ways to get it ‘right’ online, it is important that we acknowledge that teaching online is complex. For this reason, we need to learn from what is known, from our previous experiences, from student experiences, to inform our future online teaching.

This response emphasizes that taking the time to inform and iteratively re-craft online teaching is important if we are to improve our understanding of how to teach online in specific contexts. Being informed could mean drawing on different resources, such as previous practices, experiences, evaluations, and research, and by reflecting on how such resources could be applied in our teaching context (Oliver and Conole 2003). As noted in the first point of *The Manifesto*, good teaching and learning is ‘an exercise in continual re-crafting, not an adherence to entrenched notions of good practice’ (17–18). Being informed could mean applying professional judgement to reflect and act on analyses and evaluations of teaching and learning (Zwozdiak-Myers 2018) with the aim to improve online teaching practices.

If we take time to draw on resources and deliberately learn more through reflection and evaluation of our teaching, we will improve our understanding of how to teach online (Hrastinski 2021). There are many ways to get it ‘right’ online. But there are also many ways to get it ‘wrong’. We need to regard teaching online as a complex and informed practice.

### [T]he Manifesto … is a Call to Attention… (Sean Sturm)

Bernard Stiegler, in ‘Relational Ecology and the Digital Pharmakon’ (2012), reminds us that attentional spaces like that opened by teaching online have an ambivalent, or ‘pharmacological’ quality. If teaching online is perceived by many in higher education as a cure for certain ills — like, for example, a perceived decline in ‘student engagement’ or the ‘relevance’ of higher education, or the supposed ‘ineffectiveness’ of the lecture as pedagogy, or simply ‘spatial constraints’ — it is a cure that can kill … because it is so often conceived of as digitally capturing the attention of students. Bayne et al.’s *Manifesto for Teaching Online* (2020) thus calls us to attend carefully to digital attention.

#### Paying Attention

*The Manifesto* primarily conceives of digital attention as a kind of presence. Students attend, or have ‘access’ to, online classes on a ‘digital campus’, as they might attend offline classes on a ‘physical campus’. And they are asked to pay attention to their learning, which attention is monitored by their teachers and the institution algorithmically and analytically. (The word monitor appears 31 times in *The Manifesto*, and the word surveillance, 75 times.) Attention — and the attendees, but also the attenders — thus becomes subject to a cost–benefit calculus that trades in a kind of ‘hyper attention’, as Hayles (2007) dubs it: hyper- as in online, but also hyper- as in excessive.

Students and staff trade information with and about each other: students want information instantly (e.g. via access to academics or feedback against learning outcomes specified in advance); staff want information all the time (e.g. via monitoring attendance or continuous assessment) — and both want as much as they need. And this exchange of cognitive capital is facilitated by digital technology. *The Manifesto* thus reminds us that ‘we should attend to the materialities of digital education’ (Bayne et al. 2020: 19), that the mantra of digital education — and cognitive capitalism per se (Moulier-Boutang 2011) — is *attention pays*.

#### Careful Attention

But, as the 13^th^ maxim of *The Manifesto*, ‘Algorithms and analytics re-code education: pay attention!’ (Bayne et al. 2020: 59), reminds us, such paying attention is different from the ‘deep attention’ (Hayles 2007: 187) that has been the norm in education for centuries, as exemplified in the close reading (Love 2013) and ‘slow scholarship’ (Berg and Seeber 2016) beloved of the humanities. As against the vigilant attention demanded by today’s digital university, for example, the ‘multi-task[ing]’ expected of students or being ‘responsive’ required of staff (Bayne et al. 2020: 28, 23), Hayles (2007: 193) recommends a ‘synergistic combination of hyper and deep attention’. Such attention might involve digitally interactive teaching or reading digital media deeply [Bayne et al. (2020: 33, 49) call the former ‘multimodality’ and the latter ‘playful critique’].

But I would also recommend careful digital attention — not so much the ‘careful monitoring’ of learning and teaching or algorithms and analytics that *The Manifesto* proposes (Bayne et al. 2020: 89), but rather careful critique, or ‘hypercritique’ (Stiegler and Ross 2017: 390). Such critique is not only self-critical (Gasché 2007), but also ‘thinks’ — or ‘cares about and cares for’ — ‘the limits of thinking … under the condition of exosomatization’. It cares about what happens to human thinking when we outsource it to tools, be they analogue or digital, as humans by nature do (Stiegler and Ross 2017: 390). This is the true call to attention implicit in *The Manifesto*: that we who inhabit the digital university must carefully attend to how digital attention can both automate us (short-circuit our collective individuation) and augment us (generate circuits of collective individuation).

## Multimodal Stancetaking

### Manifesto as a Stancetaking Move — Performative Indexlicalization in Digital Education (Chie Adachi)

A celebrated linguist, Du Bois (2007: 163), argues that stance is ‘a public act by a social actor, achieved dialogically through overt communicative means (language, gesture, and other symbolic forms), through which social actors simultaneously evaluate objects, position subjects (themselves and others), and align with other subjects, with respect to any salient dimension of the sociocultural field’. Stancetaking, as a performative move, therefore, offers a useful theoretical framework by which to analyse and respond to *The Manifesto for Teaching Online* (Bayne et al. 2020).

The idea of ‘positionality’ is at the centre of *The Manifesto*, carefully laden with philosophical and political linguistic resources, in which ‘speakers and writers are necessarily engaged in positioning themselves vis-à-vis their words and texts … audiences (both actual and virtual/projected/imagined), and with respect to a context that they simultaneously respond to and construct linguistically’ (Jaffe 2009: 4). And this move, open to further interpretation and re-coding, works at multiple levels — individual teachers, institutions, and higher education as a sector, across places and times.

In *The Manifesto*, the use of modal auxiliaries such as ‘can’ and ‘should’ subtly implies the attitudes of the authors as social actors with hope — ‘Online *can* be the privileged mode’ (Bayne et al. 2020: 133). The positioning of what is and what is *not* the act of teaching online sets the boundaries and realigns its position with intent — ‘Distance *is* temporal, affective, political: *not* simply spatial’ (Bayne et al. 2020: 153). Socio-politically charged references provoke resistance with ideology — ‘Online courses are prone to *cultures of surveillance*. Visibility is a *pedagogical* and *ethical* issue’ (Bayne et al. 2020: 173) (emphases all mine).

**Fig. 2:**
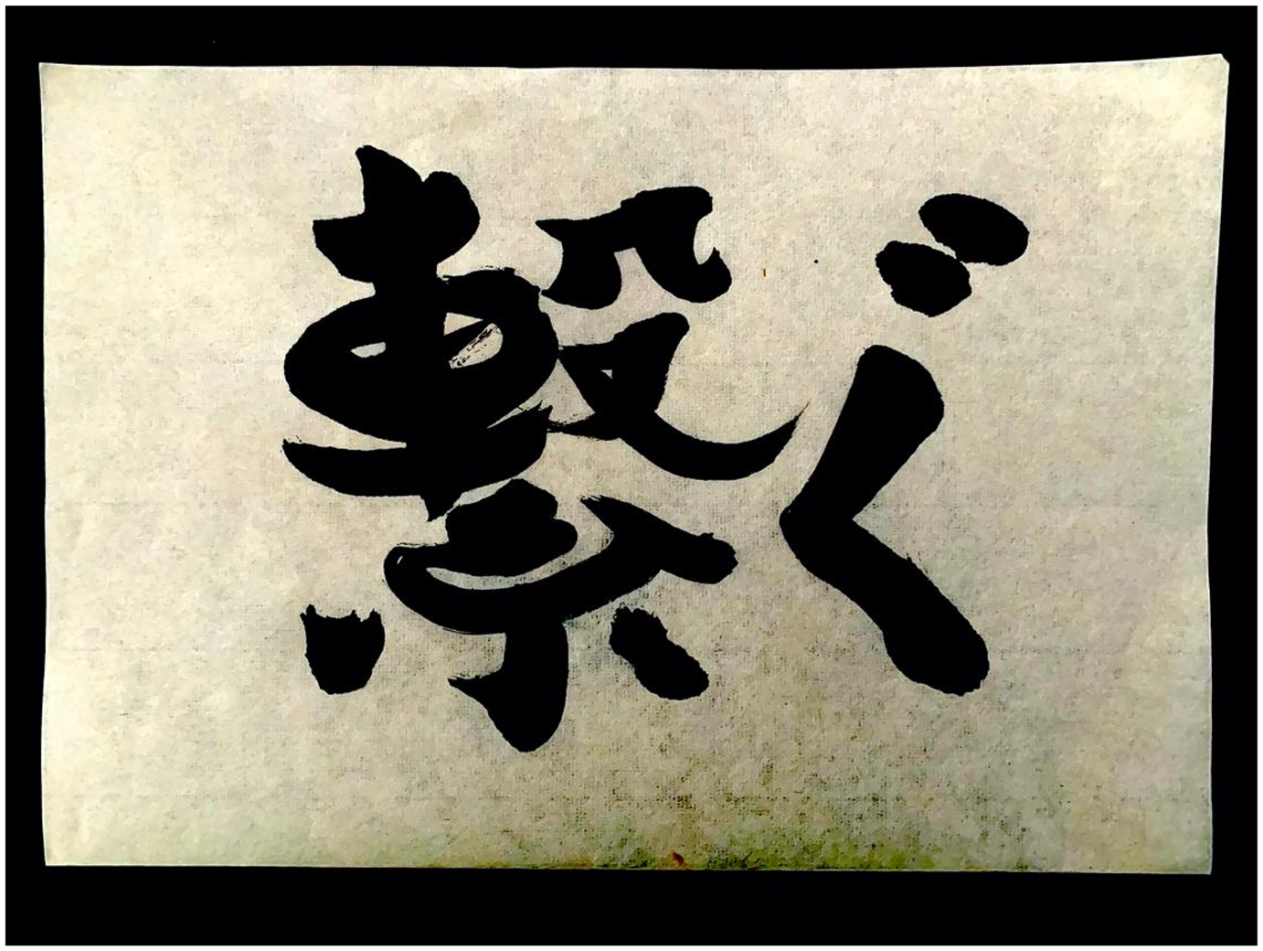
When East meets West; linguistic diversity meets representation; black blends with white; arts emerge with social science; analog delights digital

The call for responses is, therefore, an act of collective meaning-making itself, in building on the stancemaking moves that can dynamically and intentionally lead to the ‘indexicalisation’ of meaning (Ochs 1992), as a repeatable pattern that conveys significance of digital education over time. To go along with this movement, the word and image in Fig. 2 perhaps offer one response and interpretation of what digital education might mean, look, and feel like — a stancetaking move to crack open this emerging discursive space and disrupt the Western academy with an alternative. And yet again, this experimental move is vulnerably open to interpretations and sense-making processes.

繋ぐ (‘tsunagu’) is a Japanese verb, which means ‘to connect’. While one word has a limiting edge, in a form of art with its colours, strokes, and structures, it can signal much more, in which context this is to be interpreted. The original was drawn with a brush and ink on a washi paper, which was then digitally manipulated by the author.

‘Text Has Been Troubled: Many Modes Matter in Representing Academic Knowledge’ (Bayne et al. 2020: 49). *The Manifesto* continues to invite teachers to challenge the privileged, move beyond the limitations of textual repertoires, and enter into a dialogue of stancetaking and indexicalization. This response is a stancetaking move from a teacher, a researcher, and a university leader who administers digital learning innovations at a university — in short, someone who has been transformed by (digital) education.

### The Multimodal Spatial and Temporal Complexity of Online Teaching Environments (Karoline Schnaider)

Educational research is challenged when the spatial–temporal configurations of artefacts and human activities coincide (Ellis et al. 2018). This is also signalled by Bayne et al. (2020) in their *Manifesto* points for online teaching activities, underscoring digital artefacts such as multimodal technologies and sign-systems. As the transformation of human activities and artefacts are relative to the spatial–temporal conditions, they invite an understanding of when and how meaning potentials change between the technologies and the teachers' and students' meaning-making based on sign-systems (Bezemer and Kress 2016). Thus, the spatial–temporal conditions are constantly multiplied in meanings between these entities, and render a complexity to teaching and learning activities, and will be unravelled here by the multimodal layer (ML) perspective (Schnaider et al. 2020; Schnaider and Gu 2021).

The MLs have been used in learning settings to identify the connections between teachers’ and students’ meaning-making via technology use in activities and the nature of the technologies based on physical and symbolic sign-systems from five components: digital *technologies*, technologies’ *functional* and *semiotic properties*, *modes of representation*, and *activities*. The MLs complement more system-oriented approaches with semiotic details necessary to understand different circumstances in technology-enhanced learning activities.

As semiotic resources, different configurations of hardware and software *technologies* afford various integration in time and space and uptake in a wide range of social or individual activities related to their size, shape, mobility, capacity, compatibility, etc. Moreover, as spatial–temporal arrangements are visualized on the composite interface through different hardware and system and application software, accessories, layout, etc. (cf. Vigild Poulsen et al. 2018; Vigild Poulsen 2018; Zhao and Zappavigna 2018), certain sign-systems for meaning-making are made available while others are excluded, expounding manifested paradigm of choices (Djonov and van Leeuwen 2018; Vigild Poulsen et al. 2018).

Machine- and user-controlled actions and sign-making are conveyed via the *functional* and *semiotic properties* through transitions and resemiotization of sign-systems (cf. van Leeuwen 2021). These properties are spatially and temporally regulating the meaning-making by, for instance, facilitating functions such as chats that support colours and pictures to be shared between users of MOOCs.

Moreover, the spatial–temporal also transpires across the outcome of the meaning-making into *modes of representation*. For instance, when the logic of temporality governing linguistic sign-systems (e.g. writing/speech) merges into the spatial logic of visual sign-systems such as images or colour, and vice versa (van Leeuwen 2020); or when writing and speech or images demand technologies to support temporal or spatial arrangement of sequences communicated (Kress and van Leeuwen 2021). Framed by the hierarchy of spatial–temporal priorities into various *activities*, transactional synchronous and asynchronous social presence is regulated by the technologies’ affordances and different settings’ social and cultural discourses (PanMeMic 2020).

The spatial–temporal conditions are not superficial, and a comprehensive perspective is needed to understand the multimodal complexity of online teaching. The ML framework provides a nuanced description of the spatial–temporal conditions for meaning-making that eliminates online teaching only as a form of delivery to enable the expansion of digital teaching methods by recognizing the power of digital technology to transform activities.

## Distant Learning is Not, After All, so Distant

### Spatial and Temporal Distances are Illusions: What Matters in Online Learning Ecologies are Transactional Distance, Interaction, and Communication (Aras Bozkurt)

If the medium is the message and has the ability to shape and control ‘the scale and form of human association and action’ (McLuhan 1964: 9), the view of online learning spaces as learning ecologies matters in terms of its ability and capacity to facilitate learning. In opposition to the notion that physical offline/onsite learning spaces are the primary learning environments in any educational process, virtual online spaces can be viewed as effective educational environments that can be conducive to meaningful learning (Blaschke et al. 2021) by capitalizing on the transformative potential of educational technology (Pelletier et al. 2021). As learning is social, there are subjects, objects, and interactions [learning] that eventually emerge in the relationship between knowing and known (Bozkurt and Sharma 2021; Blaschke et al. 2021).

The perceived distance (e.g. temporal and spatial) between knowing and known is crucial in conventional physical offline learning ecologies, whereas in virtual online learning ecologies, distance is a relative term that is subject to learners’ interpretations. In terms of learning and learning resources (e.g. teachers and peers), what matters is not spatial or temporal distances, but the width of the communication and the depth of interaction that are defined by the learners’ self-learning needs. In addition to educational communication and interaction (Moore 1989), this view requires understanding the importance of transactional distance (Moore 2019), an idea developed from Dewey’s (1938) concept of the transaction. Accordingly, psychological, communicational, affective, and socio-cultural distances are the real barriers to meaningful learning in online learning spaces.

With its capacity to facilitate interaction and communication by eliminating the effect of spatial or temporal distance to a minimum, online distance learning does not consign learners to a diaspora. Rather, online distance learning allows learners the opportunity to search for new learning ecologies, where they can intellectually grow and flourish and ‘nurture, and sustain their connections’ by ‘cross-pollinating among multiple dimensions, paths, and layers of networked learning ecologies’ (Networked Learning Editorial Collective et al. 2021: 350–351). It is important to note, however, that these spaces need to be designed and contextualized for learning (Garrison et al. 2000) in order to generate a proper sense of learning (Rovai 2002) and to build a learning community (Wenger 1999). In effect, what we see and have in our online learning ecologies is the sum of our individual and collective decisions, a point that must be acknowledged. To explore the true potential of online learning ecologies, we need to focus on the meanings and functions of these ecologies, not on their forms.

It is also important to highlight that to create an ideal learning ecology, one that is shaped, informed, and built by learners’ self-learning needs, learners should be given agency and be empowered to explore new learning paths, encouraged to navigate in their learning quest, and equipped with skills to survive in online learning ecologies. With these, they would be better positioned to eventually find their true selves in the pursuit of information, knowledge, and wisdom.

To conclude, it would be beneficial to consider the following critical questions to better digest the above arguments: Are online learning ecologies merely synthetic structures constituted by binary codes? Does our rooted ethos (fundamental values) prevent us from unlocking the true potential of online learning and hinder us from discovering the *mythos* (interconnected knowledge) and *logos* (science) of online learning? Are physical offline learning spaces really primary and virtual online spaces, secondary? Online or offline, what makes these spaces dynamic or static? As a term, what does distance genuinely refer to? Our responses to these questions will define the value of these spaces and reposition them in the broader educational landscape.

### Distance Learning Under the Lens of Bakthinian Chronotope and Deweyan Inquiry (Chrysi Rapanta)

As stated in Chapter 17 of *The Manifesto for Teaching Online* (Bayne et al. 2020), *distance* education carries negative connotations; it conveys a sense of what is missing rather than what distance offers. The term distance education was born to refer to the actual (physical) distance between teachers and learners, as opposed to the physical proximity allowed with the traditional methods. Similarly, the initial idea of ‘open’ university was limited to this physical distance, with openness being later opened to more inclusive functions of not only everywhere but also everyone, everyhow, everywhen (Dalsgaard and Thestrup 2015; Deimann 2019). As Bayne et al. (2020) suggest, distance learning is not limited to physical distance education when this physical distance forms a by-default part of the institution’s instructional model. What is more, the ‘distance’ in learning at a distance does not simply refer to the physical distance between the learners and the teachers, but other types of distance such as temporal, affective, and political. These other forms of distance must be considered within any framework of an effective online learning environment where full teacher presence must balance the physical absence (Rapanta et al. 2020).

This idea of a *non-distant* distance learning is justified from a philosophical point of view and is not, in fact, new. Under a pedagogical philosophy of learners’ self-completeness and autonomy, learners are *always* ontologically distant: they make their own meanings in their own chronotopical contexts under their own conditions and aims. In this sense, Bakhtin’s concept of chronotope (space–time) is useful in helping us understand learners’ *always-becoming* sensemaking processes. As Steinby (2013: 116) argues, chronotopes can be understood ‘as time and place not in the physical sense but in the sense of the (right) moment for certain kinds of human action’. During a process of learner-initiated and teacher-guided inquiry, teachers must develop a deep understanding of learners’ chronotopes in the narrative sequence of learning events: when and how do learners find and make sense of the contents taught? Why does an activity make more sense than another? What are some critical incidents in each learner’s sensemaking journey? How do learners’ past, present, and future interact in each moment?

Inquiry, from a Deweyan perspective, is a lonely journey which becomes social mainly through reflection. Reflection, being a higher-order skill, is not always spontaneous — needs staging and fostering of learning experiences to reflect on. The design of learning experiences in ways that they become worthy, and springboards of reflection, is a main function of a remote teacher and maybe the only way they can have access to the learners’ individually shaped chronotopes. The more the designed experiences correspond to, and even help shape, the learners’ chronotopes, the more the distance between (physically distant) teachers and learners vanishes. Because the teacher’s making sense of the teaching experience comes to reflect the learner’s making sense of the learning experience. Teaching and learning chronotopes (self-representations of cognitive, affective, social, and other contextual aspects space–time ‘right’ moments) become the two sides of several fragile mirroring reflections in the fluid river of making sense with others and alone.

### Tele-Proximity in Infosphere and Onlife (Chryssa Themelis)

Learning about learning and learning to sail. …

It focuses on speed and the orientation of the wind. (Goodyear 2021: 3)

Education for the onlife (make a living and learn online) is a process of constant refinement to embrace technology-enhanced technologies in the transmedia landscapes and oceans. The resources are abundant and available in the infosphere (resources via Internet); so, we need to think about educational institutions, not so much as content providers but as idea-processing arenas: developing the researchers’ mindset, global values, and collective intelligence (*demosophia*) in the most unpredictable time of humanity. Goodyear (2021) talks about learning to learn and learning to navigate according to the speed and orientation of the changeable winds. Ito states that:

We need to embrace the unknowability – the irreducibility – of the real world that artists, biologists and those who work in the messy world of liberal arts and humanities are familiar and orientation of the winds and speed of change comfortable with. (Ito 2017: para 9).

Research is the key to demosophia and ubiquitous learning. To do so, we need to re-establish universities as research-based institutions. Researchers/professors could better teach students how to e-research for a living. Research to online communities could be organized according to the principles of social physics (Pentland 2014) to guide social exploration in online networks. The main principles of social physics are democratic and relational engagement with peers within courses and outside of them from a multi-disciplinary point of worldview. In short, making people search and work together towards meaningful, pragmatic, and shared goals is the path to enhance *demosophia*/wisdom of the people (Christakis 1993). *Demosophia* is the ultimate goal of academia that needs to embrace all forms of learning (formal and informal) and open all communication media (Rapanta et al. 2020) to air the voices of students and educators as life-long learners.

The challenge of teaching online is social trust, but tele-proximity could be the road ahead to resolve transactional distance implications. The biggest obstacles we need to consider are (a) how to safeguard authenticity (transparency of procedures) of institutions and (b) build social trust (against multiple identities/roles, and strategic misinformation) in the infosphere and onlife. A potential path ahead could be tele-proximity. Tele-proximity theory is an online embodiment that explains how instructors and students are connected in a synchronous networked environment via tele-operations (Themelis 2013). Embodiment is crucial to build social trust because of body language (Pentland 2008, 2010). Video conferencing and the latest version of Holographic conferencing (Themelis and Sime 2020; Adhikari 2020) offer opportunities to see each other as embodied identities. Embodiment while teaching online seems to reinforce entrusted relationships, spread habits and values (Themelis 2013) that can be reinforced by the powers of connectedness (Christakis and Fowler 2009). Proximity to the right people and democratic networks save years of education and increase creativity and innovation (Pentland 2014).

Tele-Proximity in the infosphere and onlife may be a manifesto for research centred institutions and democratic engagement in networked learning as embodied identities connected to research, share, and enhance *demosophia*.

## Campus, Citizens, and the Digital; We are the Campus

### Campus Beyond Campus (Klaus Thestrup and Tom Gislev)

#### Globeness

*The Manifesto for Teaching Online* (Bayne et al. 2020) talks boldly about globeness and going beyond a university that simply reaches out to the wider world and instead points towards a university that is imagined through strong connections across time and space (Sheail 2018). We would like to support this idea by suggesting we develop online learning and digital education in several directions simultaneously. One is in the direction of developing global and local networks that includes both physical and virtual spaces (Networked Learning Editorial Collective 2021; Networked Learning Editorial Collective et al. 2021). The screen and the camera function on a mobile device can become the connection between the different physical and digital spaces and groups of people spread out globally.

To be global, to act globally or to feel connected has to do with a return of the makerspace, the landscape, the playground, and the classroom itself in new ways in a digitalized world, where the Internet connects more and more people and places. The student and the teacher might be separated, but they are not alone in front of a laptop only receiving and discussing information. If one makerspace can be connected to another somewhere else inspiring each other, playfully transforming the objects, ideas, and thoughts of each other into something new that works locally, then the connected campus might extend to the far corners of the world and be as vibrant and alive as the original campus in one area, city or country could be.

#### The Flexible Meeting Place

We also suggest *the flexible meeting place* (Thestrup et al. 2018; Gislev et al. 2020) as a tool for connecting, producing, experimenting, and playing together. It is a way of connecting the participants in a multi-modal understanding of communication, deciding what digital and analogue tools, space, and materials to use in what way and when. It is a question of using, combining, and testing different software and hardware as long as it makes sense to the participants, and their desire to know, to act, and to unfold search processes. Technology, not being neutral, but multistable (Ihde 1990), mediates the perceptions and actions of the participants (Verbeek 2005), and by that means, co-shapes the space, the connections, and the network. It is a process to start, to develop, to maintain, and to participate in one or more meeting places that stretches all the way from using mobiles in a workshop in the suburbs to entering vast digital worlds together.

#### The Experimenting Communities

We suggest, finally, that the partners involved in networked digital online education can be understood as playful searchers in *experimenting communities* (Shumar et al. 2021; Dittert et al. 2021), where the purpose is to experiment with, and reflect upon, the processes of becoming a part of networks and combining analogue and digital spaces. Communication and production can take place while dealing with local and global challenges and fascinations. The students, teachers, and researchers are all together in a process of asking questions and searching for answers establishing a global campus.

### Ready for Online Teaching, Ready for Digital Citizenship? (Alex Örtegren)

Described as a force rapidly shifting higher education classes from on-campus to online, Covid-19 has prompted studies on the extent to which teachers are ready for online teaching (e.g. Cutri et al. 2020; Scherer et al. 2021). While such studies can contribute to the understanding of HE teaching, asking if teachers are ready (for…) might imply online-teaching readiness as a singular event or state — not necessarily a transition as much as a disruption — for which teachers are either prepared or not.

In contrast, *The Manifesto for Teaching Online* (Bayne et al. 2020) draws attention to teaching contexts constantly in flux where time and place collapse with digital technologies and complex human and non-human interactions give rise to unexpected turns. Rather than being ready for online teaching, teachers in *The Manifesto* seem to be always *in the process of becoming ready* as the teaching context never is fixated. Consequently, any attempt at establishing ‘best practice’ is destined to be inadequate, which begs the question what online teaching teachers are ready for (or ought to be), pandemic times or not.

A key *Manifesto* statement, ‘we are the campus’, echoes the fluidity above, suggesting a *we* that constitute the campus which at the same time is constitutive; the sociomaterial mesh that is HE also is us. This fluidity refuses to be confined by brick and mortar, and in its elusiveness opportunities may lie. If we are the campus, we can work to change it and, more importantly, how we ‘do’ online teaching.

HE is not just about skills and knowledge, however, but also about citizenship (Annette and McLaughlin 2005; Bryer 2014; cf. United Nations 1998). Aside from forming citizens, HE contributes to ‘critical traditions of thought which in direct and indirect ways contribute to the resources which enable us to conceptualize the notion of citizenship and bring about its flourishing in any given society’ (Annette and McLaughlin 2005: 61). Citizenship formation is thus inherent in HE.

In postdigital society (Jandrić et al. 2018), citizenship is changing as digital technologies blur the borders between the physical and the digital (Frau-Meigs et al. 2017), and interpersonal and human–machine relationships (Burbidge et al. 2020). This can be contextualized within a broad understanding of citizenship as multi-tiered (Yuval-Davis 1997), involving for instance identity and culture (Banks 2008; Osler and Starkey 2005), and as something individuals *do* rather than have (Van Gunsteren 1998/2018) where digital citizenship is an important aspect (Jørring et al. 2018; Lindgren 2017; Pedersen et al. 2018; cf. Carretero et al. 2017).

If citizenship is inherent in HE, and if citizenship is changing in postdigital society, what does this mean for HE teaching, particularly online teaching? Without providing any clear answers, one approach could be to call for attention to questions relating to digital citizenship. However, instead of asking if we are ready for digital citizenship, perhaps a more apt question would be if we are *in the process* of becoming ready.

### Reflections from the Time of Educational Closures and Openings (Eamon Costello)

As the world turned upside down in 2020, copies of *The Manifesto for Teaching Online* (Bayne et al. 2020) were furiously thumbed in search of pedagogical handholds. Debates about online teaching and all its components became critical. Terms like ‘remote emergency teaching’ (Hodges et al. 2020) may have contributed to the lexicon of online education as deficit but ultimately everything happened in the service of keeping students on their way. Campus was closed. Fortunately, we are not that campus. We are not the stones of the University. We are the people in and of it. We are the other campus. Campus was open. People were open, to ideas, to possibilities, and to creatively engaging with flipped reality.

Two particular provocations of *The Manifesto* — the entangling of openness with closures and the challenge to distance as deficit — caused me to reflect on my own educational practice. Education has many opens. For me, it has been the venerable tradition of correspondence and distance learning. To this lineage belong the thousands of distributed learners of Anna Eliot Ticknor’s 1873 Society to Encourage Studies at Home — which brought higher education to women to whom it was denied. Establishing the first correspondence school in the United States, Ticknor leveraged the technologies of the postal service and libraries to educate over 7,000 women across the country (Lee 2017). It evokes distance as the space we use to learn in and across.

Another open speaks of education as public good. Almost mundane concepts such as Creative Commons licensing help reduce friction of access to information at a basic level. If nothing else, its badges serve to remind us of the barriers, geo-locating firewalls, prestige boundaries, and privilege gaps that circumscribe and bind knowledge. Some access and equity gaps have been scored deeper during the pandemic. In fear some doubled down on prestige economies, putting their faith in price as a function of value.

But is access to knowledge, or the simple monetary price of this, even a fraction of our biggest problem? Do we look too much into ‘this fantasy of a weightless and untethered digital education?’ (Bayne et al. 2020: 13) Does the world really need more unfettered access to effusions of digital content? Is educational technology, as Selwyn (2016) harshly decried, already too ‘full of bulshit’? What if we over-share? Could we open onto unsafe epistemological areas (MacKenzie et al. 2020)? And if everything were free, easy, and open what would we struggle for or with?

Answers may lie in another open that unfolds or folds education — one rooted in the deep drivers of our teaching: open pedagogical practices. Such practices invite us ‘to be in the present, to remember that the classroom is never the same’, even when conventions may stress the opposite paradigm. It may now be precisely a time for renewal and rejuvenation of our teaching, the educational opening of our ‘minds and hearts so that we can know beyond the boundaries of what is acceptable, so that we can think and rethink, so that we can create new visions, celebrate teaching that enables transgressions—a movement against and beyond boundaries. It is that movement which makes education the practice of freedom’ (hooks 1994: 24). We are not the campus and educational openness does not depend on campus closures.

### It’s (Not) the Technology, Stupid! on the Opportunity and Challenge of Looking Beyond the Technological in Teaching Online (Gideon Dishon)

My propositions are elucidatory in this way: he who understands me finally recognizes them as senseless, when he has climbed out through them, on them, over them. (He must so to speak throw away the ladder, after he has climbed up on it.) (Wittgenstein 1961: 89)

Considering how much of the unexpected managed to cram into a single year in 2020, it is almost troubling that so many of the key insights concerning teaching online (at least in its emergency form) have already been laid out in *The Manifesto for Teaching Online* (Bayne et al. 2020). If nothing else, the pandemic has highlighted the importance of going beyond the dichotomy of online vs. face-to-face, the urgency of attending to broader sociomaterial assemblages, and the disparities of technology use across educational contexts (e.g. Reich and Mehta 2020; Teräs et al. 2020). Hence, the crisis stressed the need to overcome essentialist and instrumentalist views of educational technologies (Hamilton and Friesen 2013; Selwyn 2013), and the ensuing assumption that novel technologies will inherently improve education (Bayne 2015).

At the same time, reading *The Manifesto* in the shadow of Covid-19 exposes the tensions underlying these aspirations, illustrating that this is easier said than done. *The Manifesto* strives to support more nuanced analyses of the role of educational technologies, in which they are understood as one factor, entangled with many other features of educational contexts and processes (Macgilchrist 2021). Yet, paradoxically, scholarly work that argues against the adulation of technology is often introduced in venues centred on such issues. It would be hard to read *The Manifesto for Teaching Online* or an article from *Postdigital Science and Education* without implicitly framing technology as the central question at hand.

It has already been suggested that we have reached the point at which an emphasis on the digital could be counterproductive (Fawns 2019; Sinclair and Hayes 2019). The prevalence of online teaching during Covid-19 could prove to be a tipping point in this context; either exacerbating the essentialist and instrumentalist conceptualizations of educational technologies or opening the window (to be reached by a Wittgensteinian ladder?) for inquiries that do not fetishize the technological. Why is this the case? First, the fruition of key arguments in *The Manifesto* could serve to draw mainstream attention to the need to go beyond technological questions. Second, the unprecedented spread of online teaching implies that this is no longer a niche phenomenon catering to specific groups of learners; a shift that could unearth how the uses, meanings, and affordances of technology vary across educational settings.

In this respect, the challenge facing critical studies of educational technology is to undermine its own demarcation as a distinct field. While this is obviously an exaggeration (suited for a 500-word response), it does reflect a key dilemma: at no time has work on educational technologies been more important, and at no time has it been more urgent to shift the focus away from the technology itself, treating the digital as a ladder to be thrown after it has been climbed.

## Digital Challenges and the Darkside: Technologies and Algorithms

### Re/mix Academic Integrity with Dispositio (Michael Hoechsmann)

It is easy to get swept up in the discourse of ‘what is wrong with students these days?’ Addiction to screens, an embrace of the dopamine economy, narcissistic curation of social media platforms, a turn away from authorship, and a loss of respect for intellectual property and academic integrity (copy/paste ethic) are just a few examples of the concerns that keep professors awake at night. Universities, for the most part, carry on as though twenty-first century literacies pose a threat to authentic academic authorship. Universities commonly employ plagiarism detection software, and academic integrity policies tend to be framed around dishonesty as a common point of departure for the twenty-first century learner. In my own university, I am required to warn students of ‘prosecution’ by an academic code of conduct should they deviate from the rules. In some cases, students may be put on notice for minor offences such as the failure to duplicate an in-text citation in the bibliography at the essay’s end.

Applying twentieth century standards of academic integrity in contemporary cultural and educational conditions demonstrates a failure to recognize the profound changes of the postdigital era. Moving beyond the ‘Gutenberg Parenthesis’ (Pettitt 2007) into the postdigital conditions of Web 2.0, 3.0, and 4.0 requires an enriched theory of literacy. The term re/mix literacies refers to an assemblage of communicational competencies/practices — drawing upon a variety of modalities — that are required for full and active participation in civic, professional, and cultural spheres (Hoechsmann 2019).

As a material practice that involves rethinking and remaking from existing cultural elements, re/mix literacies support twenty-first century logics of innovation, creativity, and collaboration. They also threaten certain cultural shibboleths such as academic integrity. When we encourage learners to produce something new by reassembling existing cultural and intellectual material, students risk being charged with plagiarism. Among the effects of the copy and paste affordances of interactive, participatory media, remix and mashup are hybrid cultural forms that involve reassembly and reworking of existing ‘texts’. The spark of originality, creativity, and ‘authorship’ lies in the yoking together of already existing elements, often with some further innovation or addition.

Here, it makes sense to make a deeper dive to recuperate the departments of rhetoric developed in classical Rome and Greece, particularly the concept of *dispositio* or arrangement. *Dispositio* involves the development of arguments, requiring careful consideration of how component pieces should come together in a composition, both narratively and logically. Not all scholastic work needs to be transformed into *dispositio,* but it can be included alongside *inventio* which is the accepted practice of original research and/or discourse produced by an individual in isolation from others. Mashups and re/mixes are examples of *dispositio*, yet it can be argued that all intellectual work is always already dialogic and borne of the fruits of many voices. Taking *dispositio* seriously does not mean throwing in the towel on original authorship (*inventio*), but rather including some *dispositio* as part of the broader re/mix of scholastic production.

### Audio Connections to Recreate a Discontinuous Campus (Jackeline Bucio, Guadalupe Vadillo and Melchor Sánchez-Mendiola)

Due to the abrupt online transition that Covid-19 forced us to embrace, video has taken a protagonist role under the assumption that it is a substitute for face-to-face encounters. Painfully, we are discovering this is not true. Video not only does not substitute the physical presence; research shows that the abundance of visual stimuli can also make collaboration difficult (Tomprou et al. 2021).

In any videoconference, the camera forces us to ostensibly acknowledge the presence of the other, and to react as we do in an in-person meeting, but with some complexities added due to the pandemic lockdown: we participate not in public or neutral spaces, such as meeting rooms or classrooms, but from our own houses. We are forced to share our house life, our soundscape, and even our relationships. If we have the fortune of living in a quiet space, this is not a problem, but if we share space with other people or family members, this may become a serious issue. This response is a call to favour audio before the video. With videoconferences used as substitutes to in-person classes, we will keep chained to the screens. Instead, with audio, a more relaxed connection channel is open, and instead of being forced to keep looking at the screen, we can just imagine we are walking with students or colleagues having a conversation. Let’s try the power of audio-learning connections.

As wonderful as videoconference can be, the abuse of this tool can become a burden. With audio, we receive enough clues to reconstruct the ambiance, tone, or mood of our interlocutors, in a less threatening way than video, it requires fewer technology resources and is definitely more engaging than just text interaction: audio becomes a middle way.

If the purpose is to connect and to construct learning relationships, audio is a powerful and less invasive form of mediating the presence than video. This can be achieved by responding with personalized audio comments to an essay, only audio synchronous sessions, reading and commenting passages in brief audios, quick audio responses in forum communications, audio comment suggestions in shared documents, etc. All these low-tech strategies are high-impact ways to engage as an alternative to videoconference. Research says audio can be perceived by students as more complete and personalized feedback than only text (Bucio 2010; Voelkel and Mello 2014; Parkes and Fletcher 2017; Hast and Healy 2018). Social network audio platforms (p.e. TwitterSpaces, Clubhouse, Discord) seem to be having success favouring audio connections.

When *The Manifesto* states that ‘contact works in multiple ways’, we believe that audio acts as a ‘convivial tool’ allowing students to recreate more personal and ‘networked learning’ (Networked Learning Editorial Collective et al. 2021). Audio allows creativity and recreation vs. the invasive and mandatory videoconference used as a substitute for in-person classes. ‘We are the campus’ in every *audioverse* that is recreated. Let’s be proud of it.

### Digital Tools are for Thinking Together: Tools for Co-Creative Coordination (Greta Goetz)

I’d like to expand on two points from *aesthetics matter* in *The Manifesto for Teaching Online* (Bayne et al. 2020): ‘sociomaterial attention to the wider network of materialities’ and the importance of teachers taking control of the tools that are used.

Online teaching in the past year overwhelmingly involved the use of tools that some call malware (Balkan 2020) and others call ‘utilities governed like empires’ (Doctorow 2021). This highlights the need for serious discussion of the tools that we use and what they represent, and why, instead of automatically downloading the latest app. There is a need to develop a culture that questions digital tools and where they harm, not just enable, creative co-individuation.

In this respect, I think of Stiegler who wrote: ‘Technics challenges us and puts us into question today’ while it simultaneously presents ‘the temptation of erasing the very possibility of questioning and being put into question’ (Stiegler 2018: 36). The practice of following trends without thoughtfully questioning them, aside from being pedagogically problematic (Freire 1972: 75), are further signs of negative performativity that brings stupefaction, neglect, and paralysis (Stiegler 2018: 35).

If we agree that a thoughtful techno-symbolic milieu is one that questions how to promote co-creative coordination, in which all individuals are the producers who emit the symbols that others consume (cf. Stiegler 2014: 78, fn. 14), how can we help promote access to and awareness of the design of tools that are available (cf. Doctorow 2021), as well as take a more pedagogically critical stand?

Alternatives can be hard to find as they do not always come up in Internet searches. One example is free software, which respects users’ essential freedoms such as the freedom to run, study, and change it. ‘This is a matter of freedom, not price, so think of “free speech,” not “free beer”’ (Stallman 2021). Consideration of free software raises ethical questions, like the difference between ‘open’ and ‘free’ in licensing or restrictions on modification. I’d like to see more awareness of how we can start to lobby for having a say right down to the level of the code of the tools we use.

There needs to be further discussion about whether it is fair to expect everyone to run the same software and whether it is acceptable to use tools that come at the price of tracking, like the aggressive JavaScript in some academic sites.

In the online classroom, questions should be raised about how to select digital tools and why, whether the tools create user-centric features or lock users into systems they have no control over; whether surveillance influences free speech, and how to assemble a set of digital tools that users feel brings out the best in themselves and in others. Online teaching should pursue ‘extra’ digital knowledge to more fully support the co-creative journey (Goetz 2021).

### Don’t Throw the Educational Principles Out with the Obsolete Technology (Helder Lima Gusso)

*The Manifesto for Teaching Online* (Bayne et al. 2020) is one of the most provocative statements in the field and will stimulate debates within the area. In the team that I coordinate, we have a statement that is systematically reaffirmed when we look for the origins of the technologies we are using, and it may be relevant as a complement to *The Manifesto*.

The life cycles of technologies shorten each day. Constant changes characterize online education: the email revolution in the 1980s; online chat and the multimedia resources in the 1990s; the development of Learning Management Systems (LMS) in the 2000s; the ascension of the MOOCs in the 2010s; and social medias and apps in the 2020s are examples of the technology changes in our field. Some of them became outdated, while others were refined to survive.

Despite the frequent changes, we have observed that some of the educational principles involved in different technologies have been relatively constant. One didactic example: in the last few years, we have seen the rise of publications and apps related to *mastery learning* (e.g. Mccourt 2019; McGaghie et al. 2020). Some of the core foundations of this proposal are sequential domain of the learning objectives; demand for proficiency in each topic before making progress; students being able to move forward at their own rhythm; constant social interaction to provide formative feedbacks; and fewer lecture classes, while other instructional means (papers and recorded classes) are used to present information. Resources for *mastery learning* are already available on LMS and other apps. In addition, systematic reviews about teaching effectiveness show that this method has beneficial effects on teaching quality (e.g. Hattie 2009).

*Mastery learning* is not only an original practice, it also disrupts online education. However, it is a procedure from the 1960s and its origins were attributed to concurrent works from Bloom (1968) and Keller (1968). Those characteristics described in the last paragraph are exactly the same as proposed in the Personalized System of Instruction (PSI or Keller Plan), created in 1962 by Keller and coworkers (Akera 2017; Cândido 2017). Furthermore, Keller (1974) highlighted that the principles of PSI are presented in previous experiences, especially in the proposition of Ward (1915) to teach Arithmetic. The new *mastery learning* has already been used for over one hundred years. Digital technology has certainly changed the way this method can be used, but the same educational principles are still in practice.

In conclusion, this example about how some educational principles survived technological changes leads us to suggest a complement to *The Manifesto*: *Don’t throw the educational principles out with the obsolete technology*. Self-instruction books may not be seen frequently in the contemporary mastery systems, but the principles proposed by Keller and Bloom remain an important contribution for those who are looking for the development of online education. In that way, it is relevant that theoretical and empirical knowledge developed in the educational field be preserved from technological obsolescence.

### Postdigital Teacher Identities: Although Algorithms and Analytics Have Re-Coded Education, we Still Haven’t Paid Attention to Their Impacts on Our Teachers (Janine Aldous Arantes)

With digitizing educational settings, we make data, which enables algorithms and analytics to exist, and as such artificial intelligence and machine learning become embedded into online teaching. That is, there has been a re-coding of education over the last decade. However, what remains under scrutinized is the impacts of the *teacher* data being collected, used, and reused. With ‘eye tracking, automated online dialogue analysis, survey data from school ecosystems, log data analysis at individual and collaborative level, and visual learning analytics applied to Internet-of-Things data’ (Nistor and Hernández-Garcíac 2018: 335), there are copious amounts of teacher data networked to external sources. De-identified and anonymized, teachers’ data is either able to be re-identified due to the networking of multiple data points, or it is degraded, meaning that it produces erroneous recommendations and insights (Culnane et al. 2017). Focussing on *teachers’ data*, this response reinforces the original call for more research ‘to investigate the ways in which educational analytics might replicate and intensify deeply embedded discrimination within our societies and our institutions’ (Bayne et al. 2020: 62) by actualizing a teachers’ digital identity according to fundamental rights and legislation.

To discuss teachers’ data, this response introduces the ‘Postdigital Teacher Identity’. The Postdigital Teacher Identity builds on the work of Cheney-Lippold (2011) and Jandrić et al. (2018). It is described here as a teachers’ identity actualization that works through algorithms and analytics, and as such artificial intelligence to infer categories of identity on de-identified or anonymized data. It is actualized by positioning various digital identities within current policy, legislation, and guidelines. Notably, the teacher has limited to no control over the Postdigital Teachers’ Identity and its construction, nor how it is used or who uses it. However, the Postdigital Teacher Identity can be considered to be both capable of controlling from afar and challenges an individual’s right to privacy.

Kemp (2020) argues that these digital identities are being largely constructed via ‘concealed data practices’ that obscure how the teachers’ data may be collected, used, and re-identified in forms of long and difficult to interpret policies. When actualizing this concept through privacy legislation, Culnane et al. (2017) have demonstrated that de-identified data can be readily re-identified, stating only a ‘small number of ordinary points of information [are needed] … to identify a person’ (Culnane et al. 2017: 20). Secondly, if less data is used to construct this identity, it is considered to be ‘highly error-prone, interpretive, and in need of adjustments to perform optimally’ (Anastácio 2020: 79), because of algorithmic bias. Also known as algorithmic fairness, algorithmic bias is a fundamental component of the increasingly large numbers of personalized ‘data-driven’ insights and recommendations embedded in online learning and underpinning educational policy.

As such, we must ‘pay attention’ to how a Postdigital Teacher Identity may have impact and implication for teachers. Without considering how a Postdigital Teacher Identity has impact, intangible forms of discrimination and the fallacy that data is anonymized will continue to go relatively unscrutinized. The re-coding of the Postdigital Teacher Identity enables intangible forms of discrimination (Roberts-Mahoney et al. 2016) to be embedded by ‘[building in] pre-existing forms of bias, racial animus, and asymmetries of power’ (Stark 2019: 3) into the ‘personalized’ insights and recommendations. With individual teachers able to be re-identified, and predictive recommendations perpetuating intangible forms discrimination, we must pay much more attention to the construction, use, and reuse of Postdigital Teacher Identities.

## Critical Omissions, Critical Considerations

### A Critical Analysis of the 2011 and 2016 Manifestos for Teaching Online (Pallavi Kishore)

The 2011 and 2016 Manifestos for Teaching Online, being manifestos, only argue in favour of online education and fail to address foundational issues.

The arguments in favour of online education are based on the presumption that everybody has access to the Internet and technology. However, not all may have this access. Similarly, not all may have access to offline education either. So online and offline education are equal in this respect. However, the lack of access to the Internet and technology, and consequently to online education, is especially concerning in the case of teachers since their livelihood depends on this access. In fact, the two Manifestos for Teaching Online clearly state that ‘[o]nline can be the privileged mode’. It is, indeed, a privileged mode. This clearly shows that the Manifestos do not cater to the universal issue of access to education. Instead, the two Manifestos are based on a presumption that people already have such an access. Therefore, they are elitist, catering only to a certain segment of the world’s population.

Moreover, education has traditionally been offline. Its conversion to online is a recent phenomenon. Certainly, it serves those who cannot access offline education. However, the current growth in online education is conditioned by factors such as the Covid-19 pandemic. So, it remains to be seen if online education will become as widespread as offline education, in the long run. In any case, access to the Internet and technology will be crucial in making online education universal.

Online education may be in the form of recorded lectures, thus, entailing some amount of flexibility for both teachers and students. However, it may also require teachers to be present to answer questions live. In offline education, teaching and student interaction happen simultaneously. But recording classes and answering questions live in a separate session is more strenuous for teachers. Without live sessions to answer questions, written communication may not always be good enough for students to clarify their doubts. Moreover, written communication, as opposed to in-class interaction, involves extra work for teachers.

Online education is particularly damaging to health. Long hours of screen time are exhausting and negatively affect vision and productivity of both teachers and students.

Hence, the two Manifestos fail to address the disadvantages of online education; instead, they highlight its superiority. Thus, they are incomplete and one-sided.

Additionally, the 2011 and 2016 Manifestos for Teaching Online do not state anything much which would be different from offline education. For example, the statement that ‘[a]ssessment is an act of interpretation’ is true of offline education as well. The questions that arise about both types of education are almost, if not entirely, the same. The only difference is that the online and offline experiences are unlike, and people might have dissimilar emotions while indulging in them. It is not as if one is better than the other because each has its pros and cons.

To conclude, the idea of coming up with a manifesto for teaching online as opposed to a manifesto for teaching (offline) does not really do justice to education.

### But What Should a Teacher Do? (Mikkel Lodahl)

For the most part, the combination of a provocative manifesto and a follow-up in-depth text works well. Section 2, in particular, is full of interesting and forward-looking thoughts and reflections, showing clearly how the new materiality of digital texts – in a very broad sense – informs and changes how we should approach the production practices and output of students.

However, the book falls short in properly examining one of its central objections to how digital education has been conceived, especially by institutional administrations around the world. In Sect. 1, the statement ‘Online teaching should not be downgraded into “facilitation”’ (Bayne et al. 2020) gets a mere page and a half. This space is dedicated to lambasting ‘learnification’ as ‘de-professionalization’ of teachers, yet it fails to commit to any positive vision of what a professional teacher then *is*. The closest is this phrase on what is under-valued by too many forms of digital education: ‘subject expertise and the broader, critical and social capabilities of the teacher’.

As I was preparing to write this response, a scandal broke out about a former teacher, turned literary biographer, Blake Bailey. Bailey allegedly ‘groomed’ his 8^th^ grade students at Lusher Extension into forming close attachments to him, whom he then allegedly abused when they reached adulthood to obtain sexual favours and even commit rape. His method? Using his subject expertise — close readings of great literature — to awaken and qualify critical and social awareness in the budding teenagers (Olmstead et al. 2021).

It is, of course, absurd to suggest that either a teacher is only a facilitator or else they abuse their power. But a clearer view of how a modern, less obviously authoritative teacher role that does not sacrifice the professionalization of the job would be welcome in the new edition of *The Manifesto*. Perhaps a way forward could be to take the insights into how student production practice and output are changed by digital education and apply these to teaching practices as well? If facilitation takes over so much of teaching practice, maybe subject expertise and even some of the critical and social capabilities and functions of the teacher can be embedded in a creational practice which is easier for management to accept because it is more visible?

As an example, I have, during the recent Covid-lockdowns, produced my own, rather rambling and badly edited videos as learning materials rather than use (excellent) videos from other sources. This produces relatability between the material and the students in a way you cannot get with external learning materials. A relatability, I suspect, could be illuminated through comparison to the parsocially founded relatability afforded by interactions through social media (Stever and Lawson 2013). Perhaps this can be a part of the puzzle in defining a positive teacher role for teaching online.

### A Dark Undercurrent of Higher Education’s Possible Future (Juha Suoranta)

*The Manifesto* declares that in the post-Covid era, there ‘is likely to be a willingness to understand that teaching online can be creative, experimental, and connected in new and productive ways beyond the instrumental “needs must”’ (Bayne et al. 2020). I agree, but there are signs, too, of structural forces that online teaching will deteriorate higher education institutions as we have known them. What follows is a dark undercurrent of higher education’s possible future.

Like the rest of the educational world, my colleagues and I managed to transform our contact teaching online on terse notice during the university’s lockdown 2020–2021. Despite the circumstances, we succeeded in ‘producing’ enough degrees to fulfil the university’s promise to the Ministry of Education and Culture, the largest funder of the universities in Finland. We had proven to be good academic workers. Perhaps too good, for the success might backfire.

The University Board approved the university’s Campus Development Strategy in fall 2020, after which it could be found deep inside the Intranet. The Strategy states as follows: ‘The experience gained through the Covid-19 Pandemic highlights the need for flexible learning, and working solutions in particular, where digital and physical environments merge to support the users’ daily lives and well-being’ (Kampuskehitysstrategia 2020–2030). You do not need a degree in rhetoric to see that ‘flexible learning and working solutions’ is a euphemism for budget cuts and giving up hiring tenures. In Fleming’s (2021) words, the Strategy’s rhetoric ‘captures the dirty flipside of the edtech trend that’s transforming higher education, concealed behind a new wave of corporate buzzwords: blended learning, hybrid instruction; digital scaffolding; synchronous and asynchronous learning; micro-credentials and so-on’.

In addition, the Strategy includes this promise: ‘The University’s goal is to be carbon neutral by 2030. As part of the target, its office and teaching spaces will be reduced by 25%’ (Kampuskehitysstrategia 2020–2030). There is nothing wrong with aiming at carbon neutrality. Still, many made the math and calculated one plus one, equating that the university is planning to eliminate our faculty building; carbon neutrality being mere smoke and mirrors for cost savings under the corporate university regime.

Due to Covid-19 restrictions on campus, the university managers launched the plan knowing that we teachers and students did not occupy the university building as we did a few years ago (see Suoranta and Fitzsimmons 2017). As I write this comment, the case is still in process.

Consequently, it is possible that we will lose our office spaces, seminar rooms, lecture halls, and, more importantly, our sense of community, and will turn, perhaps, into digital nomads without any other social existence than our digital presence. Many might feel betrayed. Maybe we managed to do our job too well and won the race to the bottom? The capitalist, neoliberal university—and the world—does not seem to follow the proverb ‘the harder you work, the luckier you get’, but the reverse.

Perhaps tomorrow, we will not say that the ‘video killed the radio star’ but that the ‘digital shift’ destroyed our office spaces, seminar rooms, and community. In the future, we might not teach in the shadow of Covid-19 but will still carry on our solitary talk in the digital sphere.

### Online Teaching Demands Epistemic Fluency: Knowledge and Knowing Matters in Online Teaching (Lina Markauskaite)

In this response, I want to advance two arguments that converge on the same point: we should take different kinds of knowledge in our online teaching seriously.

**Fig. 3:**
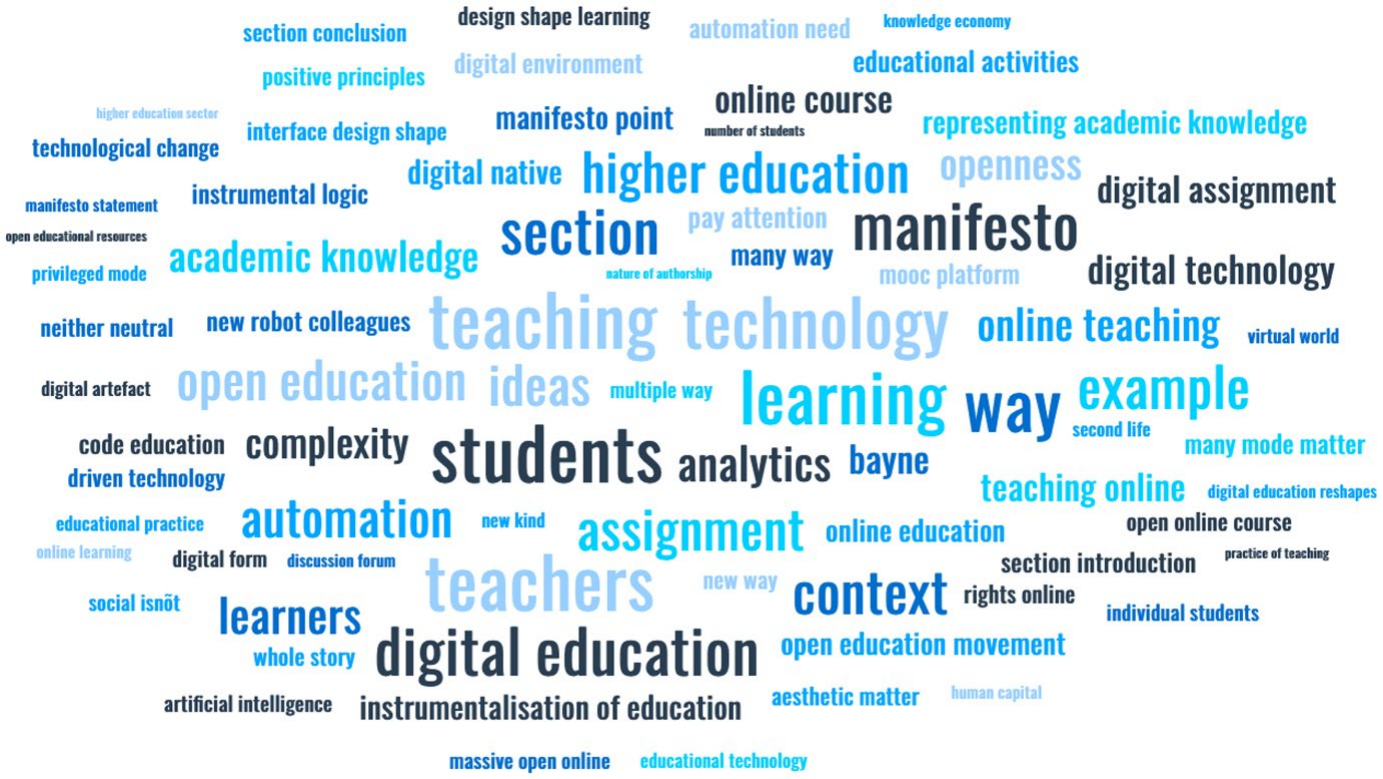
Most frequent phrases in *The Manifesto* produced with MonkeyLearn (https://monkeylearn.com/word-cloud/. Accessed 15 July 2021.)

My first concern is about what we teach when we teach online. Discussions about digital education often focus on ‘how’, ‘why’, and ‘whom’ we teach but rarely say much about ‘what’ we teach. And, when they discuss ‘what’, the most common answer is ‘academic knowledge’. The most frequent phrases in *The Manifesto* illustrate this (Fig. 3). One may argue that this is the way it should be. (In the end, universities are custodians of academic knowledge.) However, the pressing challenges of the present day—be it climate change, workplace diversity or truth decay—urge us to prepare students for working with knowledge across and beyond academic disciplines.

*The Manifesto* insightfully points out that online education offers unique opportunities to engage students in new knowledge-making practices, such as creating digital artefacts that remix and represent academic knowledge in new multimodal forms. However, my concern is that remixing academic knowledge does not necessarily result in better or more valuable knowledge. Students’ capabilities to recognize different kinds of knowledge and embrace different ways of knowing fluently and knowledgeably are critical (Markauskaite and Goodyear 2017). Therefore, when we ask students to create digital artefacts, I suggest making sure that we are asking them to create digital *knowledge* artefacts and develop their capabilities to produce valuable knowledge.

My second concern is about the ecology of human knowledge embodied in the discussions of online teaching. What do we need to attend when we teach online? *The Manifesto* does an excellent job articulating some political and philosophical ideas and shared values for those who teach online. It also insightfully notes that ‘the social isn’t the whole story’ and ‘we should attend to the materialities of digital education’ (Bayne et al. 2020). I want to push this further and argue: We should attend to the human biology and cognition.

My concern is practical. Biology and cognition have been the least popular, even undesirable, words in postdigital discussions. However, accumulating evidence about negative effects of online education on mental health and well-being leaves me with an uneasy feeling that something important has been overlooked. As Hutchins (2010: 712) argued: ‘Activity in the nervous system is linked to high-level cognitive processes by way of embodied interaction with culturally organized material and social worlds.’ Therefore, when we talk about online teaching, we should also talk about human bodies and minds. I believe it is time to embrace human biology and cognition into the same assemblage of digital education as values, policy, digital technologies, learning spaces, and voices of students and teachers.

### Beyond the Single-Student Name — a Posthumanist Response (Sara Mörtsell and Tanya O’Reilly)

As PhD students and teachers interested in digital education from a posthumanist perspective, we often encounter the discomfort and difficulty with two issues: going beyond representation and decentring the human. In an attempt to embrace and reshape these issues in this short space, we respond to reading *The Manifesto for Online Teaching* (Bayne et al. 2020) with an encounter informed by posthumanist relationality (Barad 2007; Latour 2005). While *The Manifesto* embraces a sociomaterial approach to education, this seems less evident in assessment practice. We see this in ‘The Flexible Meeting Place’, ‘Beyond words’, which rethinks authorship and meaning-making, but also struggles to move beyond representation and the central position of the human:

Considered from a sociomaterial stance the teacher might ask where, among the names associated with book chapters and journal articles, is an acknowledgement of the designers, coders, focus group participants, finance raisers and factory workers whose industry and inspiration are entangled in the web essay that bears a single student’s name? (Bayne et al. 2020: 70)

In this response, we want to examine what happens when we shift to what a single-student name on a web essay *does* rather than what it *means* and *represents*. We do this by following the signature as an actor (Adams and Thompson 2016). This means turning from the single student name to the signing practices with its many micropractices. Based on the assumption that the social and material are mutually entangled, we explore its performative enactments.

Signing enacts and is enacted by people, technology, educational norms, and discourses. The signing intra-acts with the submit button, the course registration, login to the Learning Management System database, third-party plagiarism software services, and submission conventions, in a manner that resists linear space and time, and materializes data. Signing interfaces assignment and judgement and is affective of tensions and public–private boundaries that span and join campus and online courses. It simultaneously exposes vulnerability and performance. Signing a name on an essay is not effortless nor the result of an individualized act; it involves multiple performances.

So, the question is less about which acknowledgements are omitted to put representation of authorship ‘right’, but rather what signing does and what we can do knowing this. We suggest it opens the precarious entanglement of signing, submission, and assessment in which no human is autonomous. ‘Practices of knowing cannot fully be claimed as human practices’ (Barad 2007: 185). Beyond representation in the case of assessment, as we have examined in this response, invites an acknowledgement to that effect.

### Online and Outdoor Teaching and Learning in a Time of Climate and Ecological Crisis (Jack Reed)

Outdoor and environmental education (OEE) has, across primary, secondary, and tertiary education internationally, relied almost exclusively on educating in physical proximity to others in outdoor spaces. These spaces often range from the campus grounds through to residential outdoor learning in far-away places. While acknowledging the exceptions of Smith et al. (2016) and Dyment et al. (2018), it is perhaps unsurprising that very little has been written about teaching OEE in online spaces and places. In the context of Covid-19, it was clear that more attention needed to be placed on the relationship(s) between outdoor learning and online teaching.

Enter my reading of *The Manifesto for Teaching Online* (Bayne et al. 2020: 125) and, particularly pertinent for this discussion on OEE in the tertiary context, part IV titled ‘face, space, and place’. While the sociomateriality associated with the classroom, corridor, or coffee shop need not shape a stark divide between physical and virtual spaces, when the ‘classroom’ is the local pond or copse of trees, this divide is indeed a stark one. This brought into focus the two linked arguments frequently discussed against the online mode, that is, ‘embodied co-presence and proximity are a necessary underpinning for quality education and that distance education is necessarily isolating, demotivating, and therefore of lesser quality’ (Bayne et al. 2020: 135). A level of friction emerges here as embodied co-presence has fundamentally underpinned OEE with proximity to place (e.g. Brown 2008) remaining a significant feature.

This discussion left me wondering, what place does connection to nature have within online teaching? Looking to literature (e.g. Lugg 2007; Nicol 2014a, b) in OEE, we see that planetary connection and care, sustainable living, and climate action are often explicit learning outcomes which may come to life when teaching and learning in and through the outdoors. As Shooter and Furman (2014) explain, taking learning outside can enable the interrelationships between humans and planet to be seen and felt. Linking back to the ‘shifting configuration of place’ (Bayne et al. 2020: 164), in a postdigital world marked by the climate and ecological crisis, the connection(s) between planetary health, ecological consciousness, and climate action are of the highest importance.

How can students develop tangible connections to spaces and places if their learning is not situated *in* these spaces and places? Of course, the picture I paint here is purposefully provocative and the relationship(s) between outdoor learning, climate, and ecological action, and online teaching need not be so disparate. For example, the links between the online and outdoor modes of learning may be seen in Dunn and Reed’s (2020) film about a learning episode with an Oak tree and educational apps (e.g. the Outdoor Journeys app) are making these transitions as seamless as ever. Placing a focus on the climate crisis is a welcome and important addition to *The Manifesto*. Moving forward, however, I feel we need to ask: how can online teaching and learning contribute towards developing planetary conscious and civically engaged citizens in this time of climate and ecological crisis?

### Campus Envy vs. Campus as Enclave (Ibrar Bhatt)

In conflict zones, the uproar when a campus is targeted is akin to that of when a place of worship or even a school is the object of an attack. Indeed, universities are nearly universally considered as places which ought to be kept safe from harm. Perhaps we can understand this safety as being twofold in character: (i) a physical safety, in that universities are almost sacred sites. (Many universities have religious origins and/or religious buildings attached to them.) (ii) An epistemic safety, in that a campus is a place where, according to Yale University’s iconic 1975 Woodward Report, a student must have ‘unfettered freedom’ to ‘think the unthinkable, discuss the unmentionable, and challenge the unchallengeable’ (Barnes 1976: 29).

Is, then, ‘campus envy’ inevitable for students in conflict zones? For instance—in contexts where a community’s own universities are regularly targeted, and youth, ambition, promise, and sense of place are perpetually attacked. To address this question, could campus envy, as outlined within *The Manifesto for Online Teaching* (Bayne et al. 2020), be more appropriately interpreted for students in sites of conflict and military occupation? While *The Manifesto* currently highlights the protective function of education regardless of place, a clearer articulation of this promise may be particularly necessary for students from conflict zones.

In an event held by the Society for Research into Higher Education on Monday 14 June 2021^5^, a group of university students from Palestine reflected on how their education has been affected by the various challenges they face, and how those problems have been compounded by the Covid-19 pandemic. Their everyday reality includes occupation and annexation of land, apartheid, harassment, bombing, and the active targeting of university campuses and academics (see El-Tohamy 2021; Reimer 2018). A common undercurrent was that all of them wished to reach their physical campus outside of Palestine, or return to it, as a symbolic and emotional ‘home’ for their learning. Many also feared that speaking out while back home even in a university webinar would have drastic consequences. They are far from the idealized distance learners imagined in many theories of digital education. For them, the physical campus, as a safe enclave and a place to talk freely about the plight of their people, is a reality and a hope which has been presented to them by the promises of university education, which pivoted entirely online in response to the pandemic. For such students, higher education promises a mandate for the generation, documentation, and representation of at-risk intellectual heritage and knowledge.

At the time of writing, there are some 40 ongoing armed conflicts in the world (Council on Foreign Relations 2021) and the experiences of the Palestinian students with respect to university education are bound to be echoed in other places. What kind of relationship, therefore, should such students have with their campus? How can digital education hold its promise as a space of safety, or as a safe place for exposure to new ideas for intellectual growth?

As distance-based modes of learning are being given more prominence and becoming more popularized, retaining the campus’ protective functions for all students from anywhere in the world is a challenge, and forms a necessary part of how the digital university ought to rethink its role in our post-pandemic — but not so post-conflict — world.

## Dissolving Structures of Oppression and Marginalization

### Being Culturally Responsive and Responsible is Not Optional (Cheryl Brown, Kathryn MacCallum, Cecile Ackerman, Carolyn Alexander)

*Nā tō rourou, nā taku rourou ka ora ai te ini.* | With your food basket, and my food basket the people will thrive.

Issues around access to equality and inclusivity in online learning are complex. As *The Manifesto for Online Teaching* (Bayne et al. 2020) notes, these relate to the material, the social, and the political. However, they are also explicitly cultural. The *whakataukī* (Māori proverb) above encapsulates how working together can support people to thrive and prosper.

Drawing on the sociological notion of capital as the resources needed for success (Bourdieu 1986), we are reminded that the ability to participate in online learning is not just about economic access. There are additional complex and wider issues of inequality to consider supporting learner success in terms of networks, connections, experiences, and opportunities.

Cultural capital, while a contested term (Sullivan 2002), has been expanded to online learning to explore the resources and practices which are increasingly important in determining success in the learning (Czerniewicz and Brown 2014).

Addressing inequalities online are complex challenges that cannot be simply solved through a systematic distribution of devices. In addition, inequality is relational and not necessarily compounded. We believe foregrounding approaches that support culturally responsive pedagogy will enable all students to successfully negotiate learning in online spaces.

In Aotearoa New Zealand strategies that explicitly support Māori and Pacific learners are acknowledged to benefit everyone (Bishop no date). This is supported by the Ministry of Education who published the Tātaiako, cultural competencies for teachers of Māori learners (Ministry of Education 2011), the Tapasa, cultural competencies for teachers of Pacific learners (Ministry of Education 2018), and Ka Hikitia, Māori education strategy (Ministry of Education 2021a). The latter has an action on ‘supporting online teaching and learning which includes access and educational requirements’ (Ministry of Education 2021b). This foregrounds issues of equity and access that can face online learners.

While none of these explicitly refer to culturally responsive pedagogy online there is long-standing experience in schooling contexts like the Virtual Learning Networks (VLN). Here, teachers, particularly those in rural and remote areas, support diverse learners online (Barbour and Bennett 2013). In a tertiary context, experience teaching *te reo Māori* (Māori language) online offers strategies which can be transferred to other contexts (Karaka-Clarke 2020). Values such as *Whanaungatanga* (building relationships) and *Manaakitanga* (caring) are crucial for Māori and Pacific students’ success in online learning (Blackberry and Kearney 2020). Online learning designs that foreground these explicitly in relation to Māori worldviews and values can proactively contribute to supporting inclusion (Rangiwai et al. 2020).

Being culturally responsive is no longer a ‘nice to have’. With increasingly youthful populations for Māori (median age 25 years) and Pacific (median age 23 years) people (Statistics New Zealand 2018), education must be responsive to our indigenous people. As the pandemic context has pushed learning into increasingly online spaces this is especially crucial (Ministry of Education 2021b).

### Humanizing Online Learning, the Critical Rebellion of Neoliberalism in Praxis (Ameena Leah Payne, Rebecca Bennett, and Cathy Stone)

In *The Manifesto for Teaching Online*, Bayne et al. (2020: 21) posit that digital education has a responsibility to be explicit in its critical rebellion of the ‘instrumental logics of neoliberalism and commodification’. This responsibility is heightened in the teaching of university students from non-traditional and equity backgrounds, where education can be a vehicle for social, financial, or cultural liberation (Freire 1972). We argue the tertiary digital *kyriarchy* (Schussler Fiorenza 2001) — systems keeping all forms of oppression in place — can be dismantled through an intentional focus on praxis over performativity in online teaching. Praxis privileges the development of authentic, reciprocal, and trusting teacher-student relationships over teacher performativity metrics and student academic performance scales. In privatized higher education institutions (HEIs), this praxis is (arguably, by necessity) motivated by revenue and profit margins; not by a desire to leverage tertiary learning as a means for educational and social equity. Equity students are often under-served in online environments (Stone 2017).

In Australia, Indigenous, regional/remote, first-in-family, mature age, low socio-economic status (SES), and those with reported disability are among the students demonstrating the lowest positive ratings of satisfaction and engagement; they also most commonly consider early departure from HEIs (The Social Research Centre 2021). We argue that these trends are not attributable to deficit narratives about additional pressures and educational gaps among equity student cohorts. Rather, the neoliberal service-model that underscores online university education *under-serves* minority students (Payne and Torn 2021).

On university campuses, social and cultural minorities are provided with a range of extracurricular services to support financial, social, and psychological well-being while studying. Thus, on-campus teachers can prioritize curriculum content and cognitive development. Online, university is highly individualized, focused on a subject-by-subject delivery model with little scope for extracurricular social, cultural, or pastoral support outside of class. To move beyond ‘transactional concerns’ (Bayne et al. 2020: 23) towards promoting solidarity and equity, we propose the incorporation of a ‘“global cosmopolitan” model’ (Selwyn 2007: 90) that turns the focus onto personable interactions in disembodied, digital spaces (Payne and Torn 2021). Emphasizing the human in the machine is critical in the pursuit of engaged pedagogy and activism (hooks 1994) in teaching online.

Supporting online students should be an institutional, not purely teacher, responsibility. However, in online spaces fostering rich interpersonal relationships with students is a reciprocal rebellion that stands to empower both student and teacher. We are interested in ‘creating an environment where engaged teaching can be sustained’ (Bayne et al. 2020: 165). Online teachers are equally under-served in digital spaces. The ‘learnification’ (Bayne et al. 2020: 21) discourse has served to downgrade ‘academics as proletarians’ (Arthur 2009: 442).

Within online HE, the teacher-student dyad becomes a proxy for social, pastoral, and cultural support. The key to enacting a digital pedagogy of the under-served is by providing teaching staff with the resources to support emotional responses to cognitive challenges. Fostering extracurricular, interpersonal trust challenges neoliberalism by shaping online HEIs as a community, not a commodity.

### Remixing *The Manifesto* with Design Justice Principles (Amy Collier and Sarah Lohnes Watulak)

As designers of online and hybrid learning, we have needed to confront the structures of exclusivity and white supremacy that are present in our design processes, structures that replicate exclusions hardwired in higher education at large. In our response to *The Manifesto for Online Teaching* (Bayne et al. 2020), we juxtapose selected Design Justice (DJ) principles (Costanza 2020) with *The Manifesto*’s principles to demonstrate how *The Manifesto* might explicitly centre a more intentional approach to inclusion. We invite readers to further explore Design Justice, and to consider how to engage with Design Justice principles in their online learning design processes.

*Manifesto* principle: ‘There are many ways to get it right online. “Best practice” neglects context.’ (Bayne et al. 2020: 7)

DJ principle: ‘We center the voices of those who are directly impacted by the outcomes of the design process.’ (Costanza 2020: 6–7)

*The Manifesto* invites contestations of orthodoxies that reduce online education to a set of best practices. We argue that as part of those contestations, the voices and experiences of marginalized students should be centred. It is not enough to recognize multiple ways of ‘getting it right’; we must also recognize that our approaches to design normalize the experiences and preferences of some students and marginalize others. Online design practices should seek to invite marginalized students into the design process at all stages, as well as via feedback during and after the course.

*Manifesto* principle: ‘Remixing digital content redefines authorship.’ (Bayne et al. 2020: 61)

DJ principles: ‘Before seeking new design solutions, we look for what is already working at the community level.’ ‘We honor and uplift traditional, indigenous, and local knowledge and practices.’ ‘We work towards sustainable, community-led and -controlled outcomes.’ (Costanza 2020: 6–7)

*The Manifesto* celebrates the opportunities for multimodal, remixed digital content to offer creative ways for students to represent their knowledge, thus troubling the traditional definitions of academic authorship and knowledge. From a Design Justice lens, these new forms could validate understandings rooted in communities of colour, indigenous communities, and queer communities. But remix can also erase ownership of individuals and communities as well, so as we create space for understanding rooted in marginalization communities, we must be cautious about how people in powerful positions can end up erasing the knowledge and labour of those groups.

*Manifesto* principle: ‘A routine of plagiarism detection structures-in distrust.’ (Bayne et al. 2020: 181)

DJ Principle: ‘We use design to sustain, heal, and empower our communities, as well as to seek liberation from exploitative and oppressive systems.’ (Costanza 2020: 6)

A Design Justice lens on this *Manifesto* principle explicitly names structured-in distrust as part of exploitative and oppressive systems that harm marginalized students and their communities. Carceral technologies, such as those used for online proctoring or plagiarism detection, have been shown to disproportionately target students of colour, non-binary students, and neurodivergent students. We must recognize the harms caused by these carceral technologies, advocate for our institutions to divest themselves of carceral technologies to protect our students, and design assessments that start with a goal of trusting and empowering our students.

## Conclusion (Petar Jandrić)

**Fig. 4:**
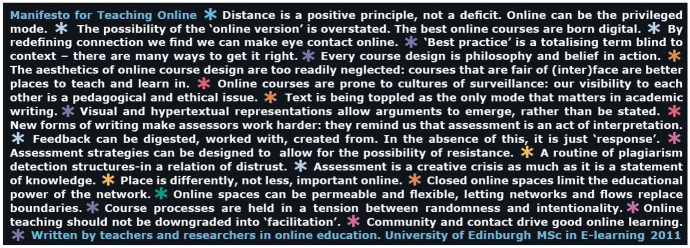
‘The Manifesto for Teaching Online’, 2011

The first version of *The Manifesto for Teaching Online* (Bayne et al. 2020) was published in 2011. It consisted of 20 simple sentences written on a simple website and an even simpler postcard (Fig. 4). In those years, I had been actively collaborating with several scholars from Moray House School of Education, and reading ‘The Manifesto’ felt almost like being with my friends in a library, café, or pub. Whole phrases, and sometimes even sentences, were so uncannily familiar. It was obvious that ‘The Manifesto’ was not written, but that it wrote itself, in talks and debates in and around the community gathered in and around the school. Taking their scholarly insights in short, punchy statements published on the web, colleagues have unapologetically exposed their own thinking and attitudes towards teaching online to widest audiences.

**Fig. 5:**
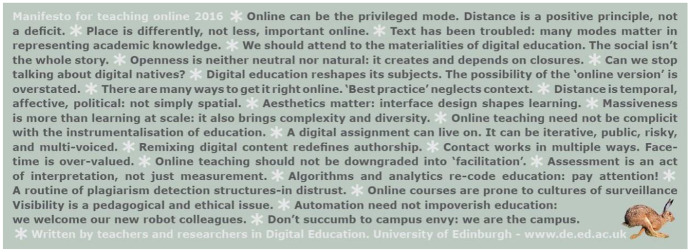
‘The Manifesto for Teaching Online’, 2016

The 2016 'Manifesto' (Fig. 5) found me in the last stages of completing my book of interviews about digital learning. In one of the interviews, I asked Siân Bayne about the purpose of ‘The Manifesto’, and she replied: ‘The “Manifesto” is designed to provoke the field of digital education practice by trying to distil some of the most interesting research findings and theoretical perspectives into punchy statements that could be used as starting points for discussion’ (Jandrić 2017: 196–197). The idea of ‘provoking the field’ resonated with me on so many levels. First, what is the field of digital education practice? The simplicity and brevity of ‘The Manifesto’ clearly implied that, for Bayne and colleagues, the field does not imply just academics, but anyone interested in these debates. Second, what is (in) a provocation? Defining their statements as starting points for discussion, Bayne and colleagues have deliberately taken the aura of academic ‘wisdom’ from their words and made their invitation more egalitarian than typical academic calls. Defined as an inspiration, rather than a solution, ‘The Manifesto’ has created an open and egalitarian power dynamic suitable for a broader-than-usual discussion.

When it was brought to my attention that the manifesto(s) were being developed into a book, I was somewhat surprised that colleagues had decided to cement their provocation into an immutable dead-tree format. Yet reading the book, I realized that it maintains the original spirit of openness. Only a few months after *The Manifesto for Teaching Online* (Bayne et al. 2020) was published, this openness has proven its value. As global education has switched to ‘emergency remote learning’ (Hodges et al. 2020), yesterday’s academic discussions have become matters of widest concern — and *The Manifesto*, which has always aimed beyond narrow academic circles, has become a hugely important resource of accumulated wisdom ready to be used in times of crisis.

During the pandemic, this accumulated wisdom and its critical perception have undergone significant transformations. Some of the provocations, such as ‘[o]nline courses are prone to cultures of surveillance: our visibility to each other is a pedagogical and ethical issue’, can now be read as straightforward prophecies. Other provocations have acquired many unforeseen and perhaps even unpleasant implications. I’m sure that, when colleagues wrote that ‘[o]nline can be the privileged mode’ — which is a statement that hovers at the very top of all versions of *The Manifesto* — they could not even imagine the cry of 27% of UK university students (and many more worldwide) unable to access their courses during the pandemic (National Union of Students 2020). I’m also sure that, when colleagues wrote that ‘[p]lace is differently, not less, important online’, they could not imagine teaching and learning from bedrooms and closets that characterized the early lockdowns.

Almost two years into the pandemic, interpreting *The Manifesto for Teaching Online* (Bayne et al. 2020) literally would be both meaningless and mean. But that does not make the provocations any less valuable. *The Manifesto* represents a specific *Zeitgeist*; immediately after the book’s publication, the *Zeitgeist* that inspired its writing is no more. At the same time, as this article bears witness, *The Manifesto* could not be more relevant today — not as a series of wisdoms about teaching and learning, but as a principle of collective sense-making, and as a message from the past that is desperately needed to build our present and future. True to its nature, in this article *The Manifesto* has made its next morph, which is one of many historical morphs, and hopefully one of many morphs to come.

Almost anyone who experienced online teaching and learning in the past two years could write their own manifesto for teaching online. Judging from recent publications in the field, I am deeply convinced that many of the sentences in these manifestos would be uncannily similar to *The Manifesto for Teaching Online* (Bayne et al. 2020). Somewhat paradoxically, the main value of Bayne and colleagues’ work is not to be found within the provocations themselves — the main strength of *The Manifesto* is in its unique approach to collectivity, its openness towards challenging our ideas and assumptions, and in laying out a clear way towards developing better ideas, approaches, and practices in the future.

I end this conclusion by paraphrasing a section from a collectively written conclusion to *Knowledge Socialism. The Rise of Peer Production: Collegiality, Collaboration, and Collective Intelligence*:

[*The Manifesto for teaching online* (Bayne et al. 2020)] is not and will never be [Bayne et al.’s] work, while, at the same time, it is and will always be [Bayne et al.’s] work. There is no [*Manifesto*], without [Bayne et al.]. And, perhaps, there is no [Bayne et al.] without [*The Manifesto*]. Or perhaps it would be better to say, there is no [Bayne et al.] in this particular configuration of the subject that is intimately constructed in and through [*The Manifesto*], if we are to take that subject as at the same time many subjects. (Gibbons et al. 2020: 303)

In our postdigital times, our knowledge of the world has never been more collective, our identities have never been more fluid, and our opportunities for collaboration have never been richer. *The Manifesto for teaching online* (Bayne et al. 2020) offers new routes for collective knowledge-making beyond narrow academic circles, and fresh opportunities for creating our desperately needed ‘new normal’. This response article shows that Bayne and colleagues’ ideas have already cast deep roots in global research community, and that they should be taken very seriously.

## Open Review 1: Online Teaching in the Epoch of Digital Reason (Michael A Peters)

It is an achievement to bring together so many scholars in on article to share their views on dichotomies between online and campus-based teaching in response to *The Manifesto for Teaching Online* (Bayne et al. 2020) and its aversion to the techno-corporate normalization of education. In another way, this collective review is an innovation and protest against the same techno-corporate standardization of scholarship based on an individualist-competitive industrial model of academic publishing following strict bibliographic guidelines that suits publishers more than authors or readers. The platform requirements of surveillance academic publishing will become even more demanding. I favour the form of collective scholarship and developed it in *Educational Philosophy and Theory*^6^ as a means to overcome the neoliberal stranglehold of scholarship (see Peters et al. 2021).

This collective review not only provides for diversity but also acts as a vehicle for consensus formation in the field. Covid-19 in 2020–21 has provided a critical test bed for online teaching and new digital technologies in education. The review is timely but also useful in responding to the *Manifesto* published a decade ago. On the whole, the lessons of the *Manifesto* have been taken to heart – an accepted emphasis on contingency, multimodality, and integration of teaching with a strong accent on best practice, criticality, and the ‘rich possibilities’ as well as the surveillance control of platform capitalism. Most contributions are autobiographically anchored in current practice and experiment and there is an appeal to a diverse set of theory focused on new digital technologies (Dewey, Bakhtin, McLuhan, Wittgenstein, Freire, Stallman, Stiegler). In particular, I appreciate what I interpret as a form of epistemological contextualism which can accommodate new developments in theory that acknowledges AI, complexity theory, and algorithmic knowledge capitalism in the epoch of digital reason.

Education is increasingly marked by two emergent and profound developments that have already begun to determine its future shape and major theoretical preoccupations: the ecological turn and the digital turn. At the most basic level, the ecological approaches in education share an ontology of interconnectivity with the new digital technologies and together decentre the individual learner redefine the student as part of larger living and technological systems. Contributors show an awareness of this historical process and how the intermeshing of these two systems leads to new possibilities for online teaching practice. There are biological and social implications arising from our growing fusion with the digital world especially with auto-poietic or self-producing new technologies that presents the teaching–learning ecosystem as a living system with its institutional, autonomous, and evolutionary transhuman visions sometimes referred to as the technological singularity.

One of the strengths of this article collection is that it refers to a common source to embrace a wide variety of conceptions of digital citizenship, ‘postdigital society’, and ‘identity cultures’ linking with extant educational philosophy and theory to emphasize the possibilities of education as a digital public good and symbolic co-production, as well as the technological dark side of algorithms that generate new problems with misinformation, plagiarism, surveillance, measurement and testing, and the use of personal learner data. There are critical contributions – ‘Critical Omissions, Critical Considerations’—also that question the focus on online teaching and education which ‘fail to address foundational issues’ or ignores ‘structural forces that online teaching will deteriorate higher education institutions’. Yet most objections seem encouraging of the *Manifesto* and seek to supplement it with sociomaterial accounts that take into account larger economic and societal forces while others, in addition, seek to dissolve ‘Structures of Oppression and Marginalization’ that emphasizes cultural responsiveness and greater humanization especially against current neoliberal policies.

If the aim was ‘to provoke’ as both Bayne and Jandrić point out then the *Manifesto* has done its job, and the collective article in the space of one collective has achieved the impossible. For this reviewer, the collective piece is an important contribution well-suited to its review function.

## Open Review 2: We are Not Stones, But We are Not Data: Postdigital, Postpandemic Education (Lesley Gourlay)

The title of this rich set of responses begins with ‘Dissolving the Dichotomies between Online and Campus-Based Teaching’. There is an assumption here both that a dichotomy exists, and also that there is a need for it to be dissolved. The contributions in various ways probe, challenge, and interrogate this notion, and a wealth of related points flowing from the abundant source of ideas provided in the *Manifesto*.

The responses are diverse in their focus, and it is beyond the scope of these 500 words to do justice to all. However, interwoven concepts surrounding both disjunctions-from and entanglements-between run like threads through much of the piece. Many provide a riposte to the notion that the dichotomy is overstated, and can be simply overcome with the right pedagogic approaches. Despite the impressive response internationally to the pandemic in terms of emergency remote teaching, two dangers might be highlighted here. The first is the resurgence of persistent utopian discourses surrounding the digital, in particular, the claim that the embodied, ephemeral, co-present life of the campus can be replicated unproblematically online. The second is a related collapse into a discourse of inevitability, which seeks to justify a wholesale move to remote digital education, on the basis that this was (allegedly) fully achieved during Covid-19.

While the successes of the pandemic period must be celebrated, I echo voices within this piece which interrogate the non-educational motives driving governments and institutions to pursue this avenue. Other responses raise cogent and richly referenced points around variously: surveillance and algorithmic cultures, inequalities, the need for criticality, the role of the margins, techno-solutionism, and learnification. The need to recognize the more-than-human nature of digital education is reiterated, with insights offered into the nature of attention, presence and distance, multimodality, semiotic resources, digital literacies, the nature of authorship, the role of audio, the centrality of space, teacher identities, and the difference between what is ‘open’ and what is ‘free’.

The *Manifesto* is characterized by Adachi as a *stancemaking* move, which I found a highly compelling way of thinking about not only the book, but theory and practice more broadly, in that a stance involves both moments of stillness and reflection, but also necessarily fluidity, movement, and alterity. I was also struck by how Rapanta refracts *The Manifesto* through Bakthin’s *chronotope*, giving theoretical purchase on the often-neglected dimension of time. However, perhaps for me, the most resonant and generative phrase was Costello’s apparently simple ‘we are not the stones of the university’. Indeed, we are not. But what this collective ‘hive’ response offers is multiple insights into how we are also not merely data, texts, numbers, or flickering images in screens. The calls of *The Manifesto* and these replying voices reinscribe the multiple, embodied, and unfolding nature of digital education, and this dense, thought-provoking piece opens up critical and illuminating avenues for our field to trouble ‘easy’ assumptions, and deepen our understanding of whatever the multiple ‘new normals’ might be, in the messy complexities of postdigitial and postpandemic education.

## Footnotes

1 Earlier versions of the *The Manifesto for Teaching Online* can be found at https://blogs.ed.ac.uk/manifestoteachingonline/ (Accessed 15 July 2021). Given that previous versions of *The Manifesto* are fully included in the 2020 book, all references to *The Manifesto* in this article point towards the latest and the most complete version.

2 See https://www.springer.com/series/16439. Accessed 15 July 2021.

3 See *Postdigital Science and Education*, *2*(3), dedicated to the pandemic challenge, https://link.springer.com/journal/42438/volumes-and-issues/2-3. Accessed 15 July 2021.

4 See https://www.research.ed.ac.uk/en/organisations/centre-for-research-in-digital-education. Accessed 15 July 2021.

5 ‘Studying While Under Occupation, Apartheid, And Pandemic: Lessons for The Digital University’ was an online event organized by the writer under the Society for Research into Higher Education, on June 14th, 2021. See: https://srhe.ac.uk/civicrm/?civiwp=CiviCRM&q=civicrm/event/info&reset=1&id=560. Accessed 15 July 2021.

6 See https://www.tandfonline.com/toc/rept20/current. Accessed 13 September 2021.
